# Mesenchymal stem cells ameliorate hyperglycemia-induced endothelial injury through modulation of mitophagy

**DOI:** 10.1038/s41419-018-0861-x

**Published:** 2018-08-06

**Authors:** Wuzheng Zhu, Yujia Yuan, Guangneng Liao, Lan Li, Jingping Liu, Younan Chen, Jie Zhang, Jingqiu Cheng, Yanrong Lu

**Affiliations:** Key Laboratory of Transplant Engineering and Immunology, NHFPC; Regenerative Medicine Research Centre, West China Hospital, SichuanUniversity, Chengdu, People’s Republic of China

## Abstract

Mitochondrial dysfunction and excessive mitochondrial reactive oxygen species (ROS) are fundamental contributors to endothelial injury in diabetic states. Mesenchymal stem cells (MSCs) have exhibited an extraordinary cytoprotective effect that extends to the modulation of mitochondrial homeostasis. However, the underlying mechanisms have not been clearly defined. Emerging evidence has suggested that mitophagy could counteract mitochondrial-derived oxidative stress through the selective elimination of impaired or dysfunctional mitochondria. Therefore, we investigated whether MSCs could ameliorate high-glucose-induced endothelial injury through the modulation of mitophagy. We observed that exposure of human umbilical vein endothelial cells (HUVECs) to high glucose triggers mitochondrial impairment with excessive mitochondrial fragmentation and ROS generation, loss of membrane potential and reduced ATP production. Furthermore, mitophagy was blunted upon high glucose insult, which accelerated dysfunctional mitochondrial accumulation, initiating the mitochondrial apoptotic pathway and, eventually, endothelial dysfunction. MSCs treatment notably attenuated these perturbations accompanied by an enhancement of Pink1 and Parkin expression, whereas these beneficial effects of MSCs were abolished when either Pink1 or Parkin was knocked down. In aortas of diabetic rats, defective mitophagy was observed, which coincided with marked mitochondrial dysfunction. Ultrastructurally, RAECs from diabetic rats revealed a significant reduction in autophagic vacuoles and a marked increase in fragmented mitochondria. Importantly, the infusion of MSCs restored Pink1/Parkin-mediated mitophagy, ameliorated mitochondrial dysfunction and attenuated apoptosis in endothelial cells in diabetic rats. These results suggest that MSCs may protect endothelial cells from hyperglycemia-induced injury by ameliorating mitochondrial dysfunction via Pink1/Parkin –mediated mitophagy

## Introduction

Diabetic vasculopathy that affects both the micro- and macrovasculature is the leading cause of morbidity and mortality in diabetic patients^1^. Diabetes-specific microvascular disease is a causal factor of blindness, renal failure and nerve damage^2^. Diabetes-accelerated macrovascular disease affecting arteries contributes to myocardial infarction, stroke and limb amputation^3^. A considerable amount of evidence has demonstrated that hyperglycemia-induced overproduction of reactive oxygen species (ROS) is the main culprit for these diabetic vascular complications, which begin with the initial development of vascular endothelium dysfunction, characterized by increased oxidative stress^4,5^. Furthermore, mitochondria are known to be the principal resource of ROS in hyperglycemic conditions^6^ and oxidative stress is inseparably linked to mitochondrial dysfunction since mitochondria are both generators and targets of ROS^7^. These findings led to the unifying hypothesis that the pathogenesis of hyperglycemia-induced endothelial injury may involve mitochondrial dysfunction and, consequential mitochondrial ROS (mtROS) overproduction.

Recently, mitophagy has emerged as an important cytoprotective mechanism for limiting mitochondrial-derived oxidative stress and preventing apoptosis^8^. However, under pathological conditions, especially in diabetes mellitus, excessive fission occurs, and mitophagy is disturbed, leading to drastic mitochondrial fragmentation, ROS overproduction and the accumulation of dysfunctional mitochondria^9^. In turn, the increased ROS further accelerates mitochondrial oxidative damage and cytochrome C (Cyt C) release to the cytosol, which amplify apoptosis^7^. Thus, targeting the improvement of mitophagy may be a promising therapeutic treatment for endothelial oxidative damage in diabetic states.

Mesenchymal stem cells (MSCs) are one of the most important multipotent adult stem cells, and have exhibited great promise as a potential therapeutic strategy due to their capacities to counteract autoimmunity, modulate the microenvironment and secrete various cytokines^10^. A wide variety of studies in animals^11,12^ and clinical trials^13,14^ have shown the exciting therapeutic effects of MSCs in the treatment of various diseases, such as ischemic diseases, neurodegenerative disease and diabetic vasculopathy. A study in diabetic mice has shown that the injection of MSCs can restore vasculogenesis and blood flow in an ischemic hind limb^15^. Co-culture of islet endothelial cell with MSCs can prevent oxidative stress-induced apoptosis, eNOS inhibition and VCAM elevation^16^. A recent study found that local transplantation of MSCs improved cutaneous wound healing via promoting endothelial cells angiogeniesis, during which VEGF-paracrine secreted from MSCs plays a central role^17^. Furthermore, our previous study also indicated that MSCs-derived conditioned media can ameliorate diabetic endothelial dysfunction by promoting mitochondrial biogenesis^18^. However, the precise endothelial targets responsible for the observed cytoprotective effects of MSC therapy have not been identified, and thorough mechanistic studies remain to be explored. Thus, with this background, this study was designed to evaluate whether MSCs can ameliorate HG-induced endothelial injury through the mitophagy pathway, a mitochondrial quality control mechanism that is distinguished from mitochondrial biogenesis.

## Result

### HG induces mitophagy inhibition, mitochondrial dysfunction and apoptosis in HUVECs

**Fig. 1:**
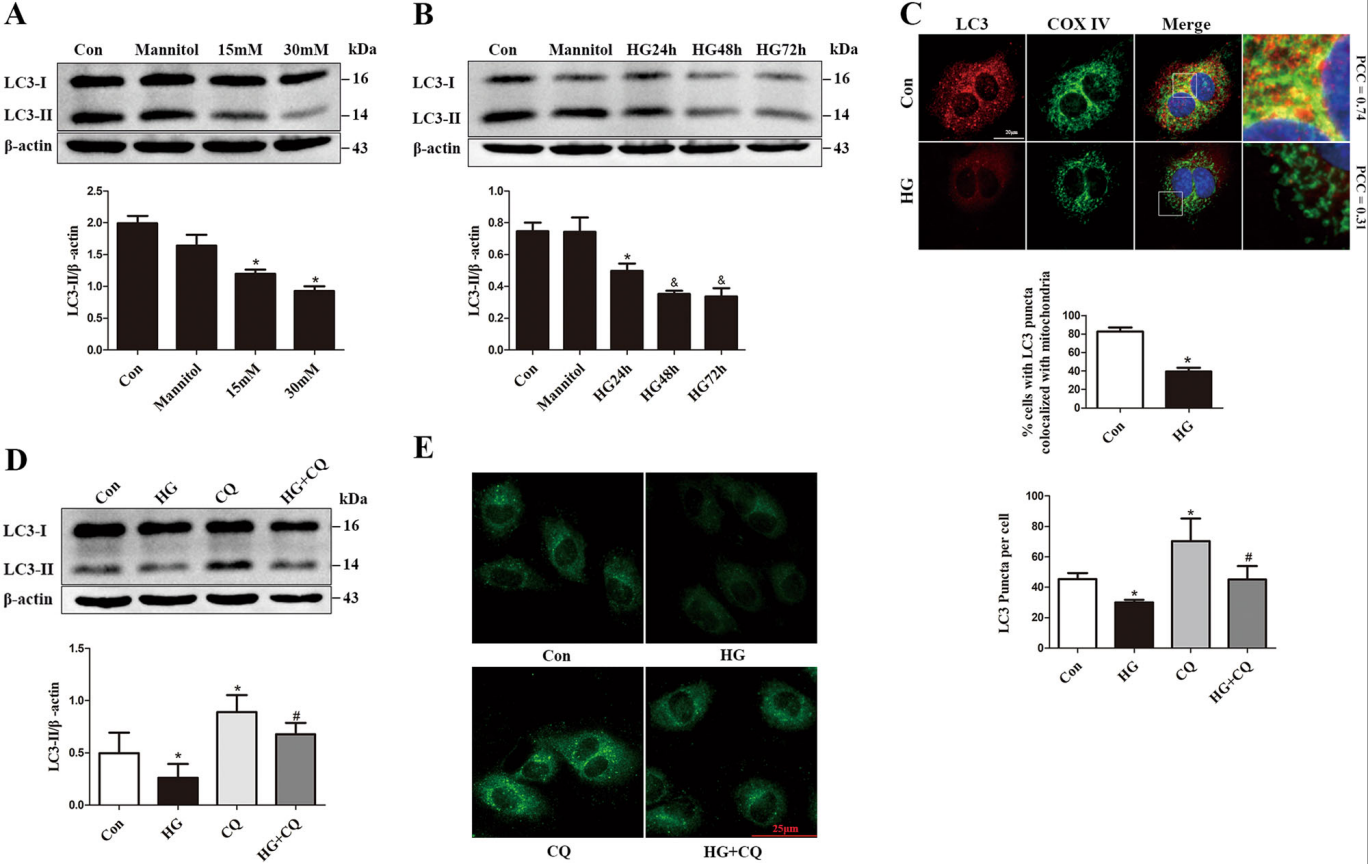
HG induces mitophagy inhibition in HUVECs. **a** HUVECs were treated with different concentrations of glucose for 72 h, and cell lysates were analyzed by Western blot using an antibody against LC3B. **b** HUVECs were exposed to HG (30 mmol/L) for indicated times, and LC3-II protein levels were detected by Western blot. **c** Colocalization between LC3 and COXIV was used as a measure of mitophagy, Pearson’s correlation coefficients (PCC) > 0.5 signify detection correlation. **d** HUVECs were treated with HG for 72 h in the presence or absence of chloroquine (CQ, 10 μM); CQ was added 8 h before the expression of LC3-II in cell lysates was determined by Western blot. **e** Immunofluorescence staining and quantitative change of LC3, HUVECs were treatedwith HG and CQ as described in **d**. Data are shown as the means ± SD from three independent experiments. (*P < 0.05 vs. Con, ^#^*P* < 0.05 vs. CQ, ^&^*P* < 0.05 vs. HG24 h)

We first determined whether HG alters mitophagy in HUVECs. HUVECs (Supplementary Figure S1) were cultured in ECM-2 medium with different concentrations of glucose (5, 15 and 30 mM) for 72 h. Mannitol was induced to exclude the effect of hyperosmolarity. Exposure to HG decreased the protein levels of the autophagy marker LC3-II in a dose-dependent manner (Fig. 1a), but increased the protein levels of P62 (Supplementary Figure S2A). Furthermore, a time-dependent reduction in LC3-II biosynthesis was observed at 24 h when cells were treated with 30 mM glucose, and this effect persisted throughout the study period (Fig. 1b). Conversely, exposure to 30 mM HG for 48 h or longer resulted in markedly increased expression of P62 (Supplementary Figure S2B). Colocalization of LC3 and COXIV was evaluated to delineate mitophagy. This colocalization was reduced under HG conditions, compared with control (Fig. 1c). To explore the mitophagy further, we evaluated the autophagic flux. HUVECs were treated with HG in the presence or absence of lysosomal inhibitor chloroquine (CQ), which blocks the degradation of autophagic vacuoles. CQ led to a clear accumulation of LC3-II protein under normal glucose conditions, but this effect was attenuated by HG treatment (Fig. 1d). Consistently, an additive increase in LC3 accumulation was not observed by immunostaining in the CQ-treated HG group compared with the CQ-only group (Fig. 1e), indicating that autophagic vacuoles were reduced and autophagic flux was blocked by elevated glucose levels.

We next investigated the mitochondria fitness under HG conditions. Upon shifting to HG concentrations, HUVECs displayed increased mtROS levels in parallel with excessive mitochondrial fragmentation (Supplementary Figure S2C, D). To quantify changes in mitochondrial fragmentation in HUVECs, we determined the aspect ratio (AR) and form factor (FF) of mitochondria in each group. Both parameters from the HG group were significantly reduced when compared with those of the control group. Consistently, Western blot analysis revealed up-regulation of the mitochondrial profission protein Drp1, whereas no significant alteration was observed in the protein levels of Fis1, MFN2, and OPA1 (Supplementary Figure S2E, F). In addition, a collapsed mitochondrial membrane potential (Ψm) was seen under HG conditions (Supplementary Figure S2G), and more apoptotic cell death was induced (Supplementary Figure S2H).

### MSCs improve mitophagy and ameliorate mitochondrial dysfunction and ROS overproduction

**Fig. 2:**
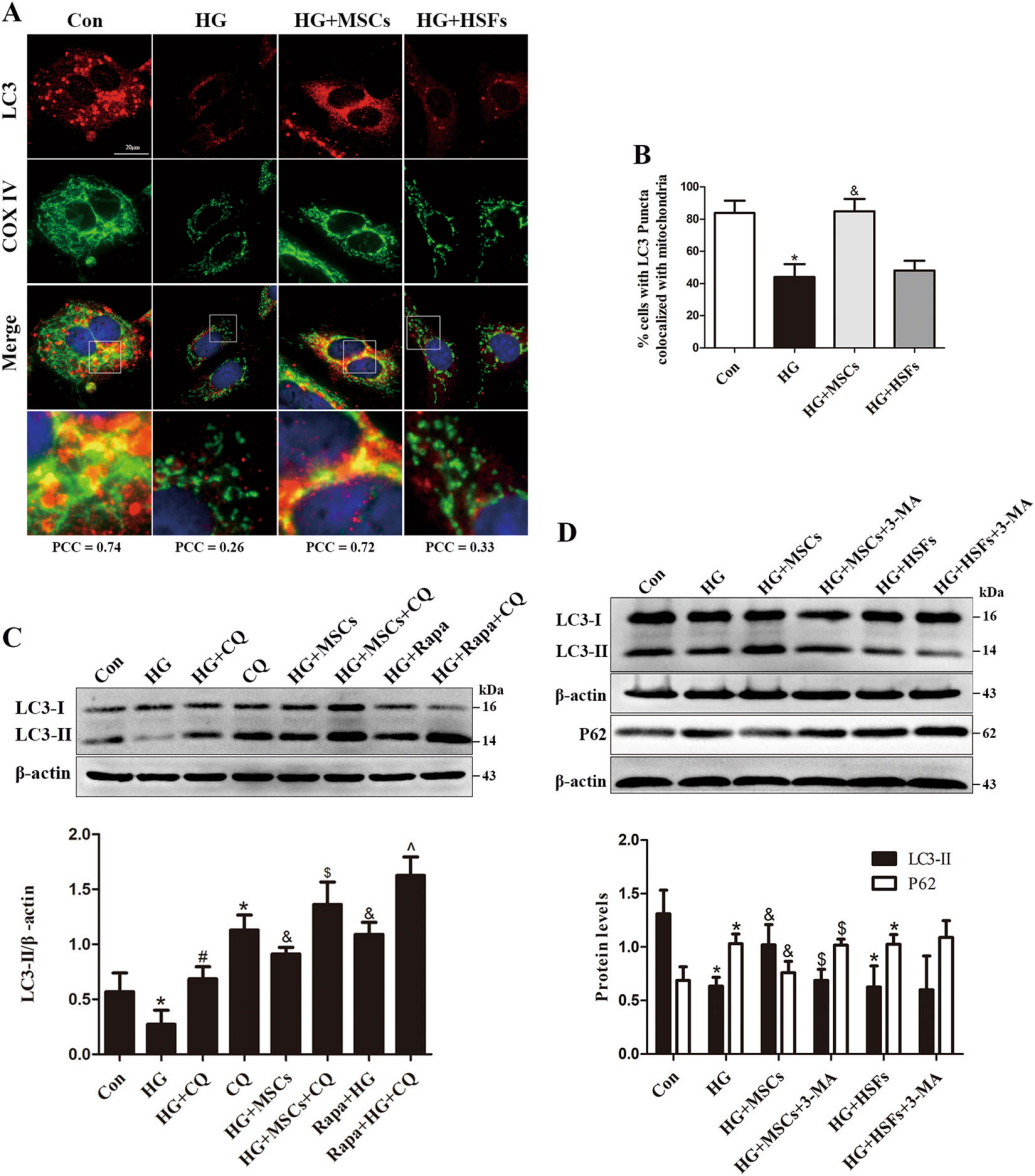
MSCs ameliorate HG-induced mitophagy inhibition. **a**, **b** Colocalization between LC3 and COXIV was used as measure of mitophagy in the different groups, PCC > 0.5 signify detection correlation. **c** HUVECs were treated with HG, HG/MSCs or HG/rapamycin for 72 h; rapamycin (Rapa, 15 nM) was administered as a positive control to induce autophagy; and CQ was added 8 h before determining the autophagic flux. **d** HUVECs were pretreated with 3-MA (5 mM) for 4 h and then co-cultured with MSCs or HSFs in HG for 72 h, and the expression of LC3-II and P62 were detected by Western blot. Data are shown as the means ± SD from three independent experiments. (*P *<* 0.05 vs. Con, ^#^*P* *<* 0.05 vs. CQ, ^&^*P* *<* 0.05 vs. HG, ^$^*P* *<* 0.05 vs. MSCs/HG, ^**^**^*P* *<* 0.05 vs. Rapa/HG)

Growing evidence indicates that mitophagy plays an important protective role in resistance to mitochondrial dysfunction-induced injury in disease states^19,20^. Immunofluorescence analysis revealed that MSCs treatment preserved the colocalization of LC3 and COXIV, which was reduced under HG conditions (Figs. 2a, b). Inconsistently, co-culture of HUVECs with HSFs did not restore this colocalization. To examine whether MSCs-preserved mitophagy was functional or completed, we determined the autophagic flux with lysosomal inhibitor CQ. MSCs administration reversed the reduction in LC3-II protein levels in HG conditions, and this effect was further elevated by CQ treatment. Furthermore, treatment with rapamycin (Rapa), a robust inducer of autophagy, significantly prevented HG-induced reduction of LC3-II protein levels, and the magnitude of this improvement was comparable to that induced by MSCs (Fig. 2c), suggesting that MSCs treatment restores the autophagic flux in a HG environment. Conversely, when HUVECs were co-cultured with HSFs under HG conditions, no apparent enhancement of LC3-II expression and reduction of P62 levels were observed in comparison with that of HUVECs co-cultured with MSCs. Moreover, MSCs-mediated LC3-II accumulation and reduction of P62 levels were attenuated by administration of autophagy inhibitor 3-MA (Fig. 2d). These data collectively suggest that this restoration of autophagic flux in HG environment could be specifically attributed to MSCs.

**Fig. 3:**
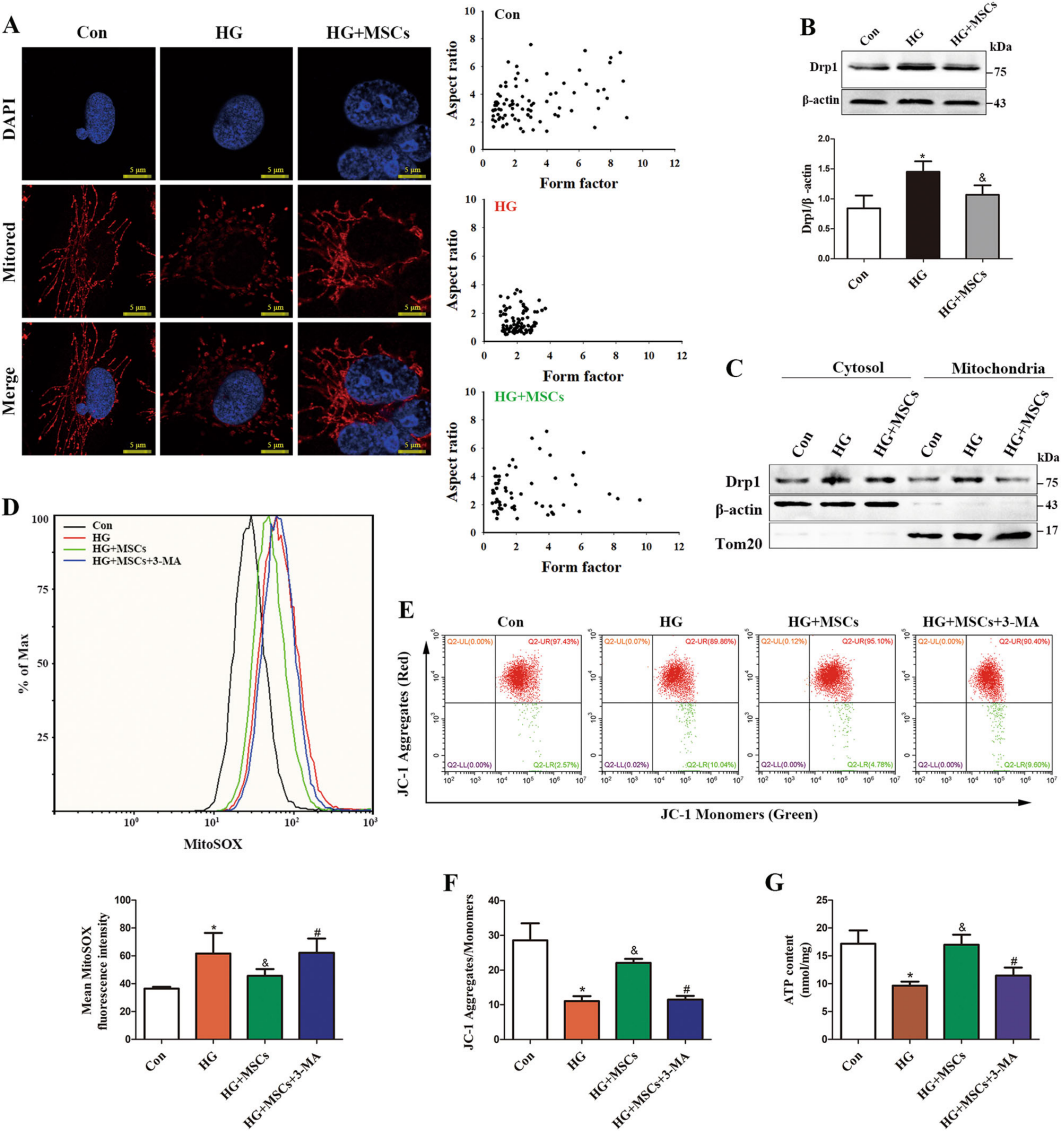
MSCs attenuate HG-induced mitochondrial dysfunction. **a** Micrographs of mitochondrial morphology visualized by MitoTracker red staining of HUVECs. **b** Western blot analysis of Drp1 in HUVECs. **c** Western blot analysis of Drp1 protein levels in cytoplasmic and mitochondrial fractions. Tom20 and β-actin were used as mitochondrial and cytosolic markers, respectively. **d** mtROS measurements described as relative mean fluorescence intensity of MitoSox Red. **e**, **f** Mitochondrial membrane potential was measured by flow cytometry. **g** ATP levels were quantified in HUVECs. Data are shown as the means ± SD from three independent experiments. (*P *<* 0.05 vs. Con, ^&^*P* *<* 0.05 vs. HG, ^#^*P* *<* 0.05 vs. MSCs/HG)

Mitochondrial fragmentation has recently been found to be an important contributing factor to ROS overproduction under HG conditions, in which it causes deleterious vascular cell signaling and subsequent endothelial dysfunction^21,22^. Thus, to uncover whether MSCs treatment could prevent HG-induced mitochondrial fragmentation, we examined the mitochondrial morphology through Mitotracker red staining. Under physiological conditions, mitochondria were elongated and filamentous, but they underwent extensive fragmentation after exposure to HG conditions. Importantly, the fragmented phenotype induced by HG was reversed when HUVECs were co-cultured with MSCs (Fig. 3a).

Drp1 is a key component of the mitochondrial fission machinery, which plays a central role in mediating mitochondrial dysfunction in HG environments, we next tested whether the inhibitory effect of MSCs on mitochondrial fragmentation involved the suppression of Drp1. We observed that, although there was a marked increase in Drp1 expression in HG-treated HUVECs, administration of MSCs significantly abolished this increase (Fig. 3b). Furthermore, MSCs treatment effectively inhibited HG-induced mitochondrial translocation of Drp1 (Fig. 3c), which is required for mitochondrial fission^23^. Notably, simultaneous analysis of mtROS (Fig. 3d), Ψm (Figs. 3e, f) and ATP production (Fig. 3g) revealed that HUVECs co-cultured with MSCs had lower mtROS, more normalized Ψm and higher ATP content than were observed in the HG group, suggesting an overall improvement of mitochondrial function. However, these mitochondrial protective effects exerted by MSCs were attenuated by the addition of 3-MA, indicating that mitophagy may participate the improvement of MSCs on mitochondrial fitness.

### Pink1/Parkin-mediated mitophagy is essential to the improvement in mitochondrial fitness mediated by MSCs in HG environments

**Fig. 4:**
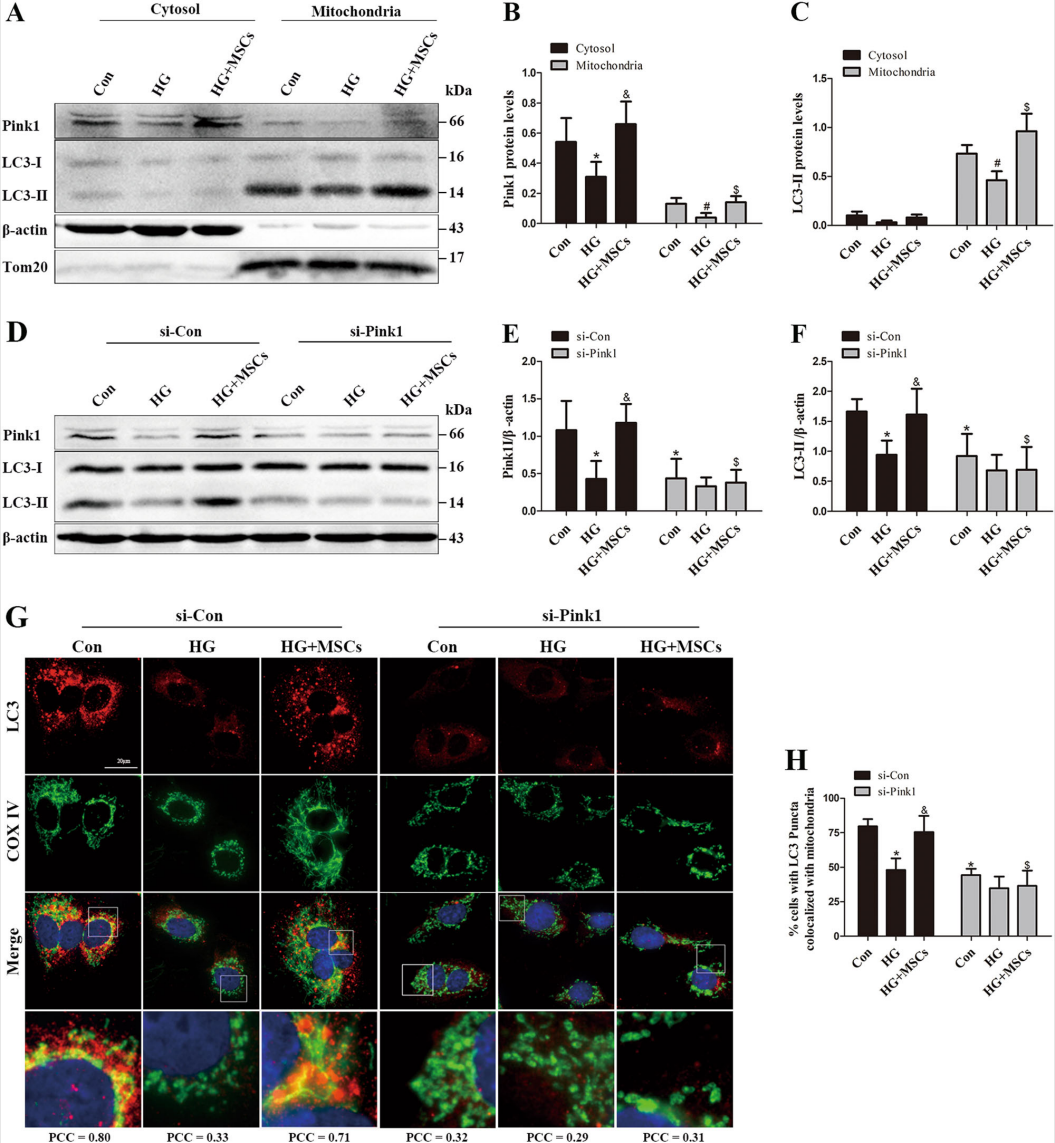
MSCs mitigate HG-induced inhibition of mitophagy through up-regulation of Pink1. **a**–**c** Cytoplasmic and mitochondrial fractions from HUVECs were analyzed by Western blot, and the fractions were probed for Pink1 and LC3 (*P *<* 0.05 vs. Con/Cytosol, ^&^*P* *<* 0.05 vs. HG/Cytosol, ^#^*P* *<* 0.05 vs. Con/Mitochondria, ^$^*P* *<* 0.05 vs. HG/Mitochondria). **d**–**h** HUVECs were transfected with control siRNA (si-Con) or Pink1 siRNA (si-Pink1) and stimulated with HG for 72 h. Pink1 and LC3-II expression was measured by Western blot. Colocalization between LC3 and COXIV was used as measure of mitophagy, PCC > 0.5 signify detection correlation. Data are shown as the means ± SD from three independent experiments. (*P < 0.05 vs. Con/si-Con, ^&^*P* < 0.05 vs. HG/si-Con, ^$^*P* < 0.05 vs. HG/MSCs/si-Con)

Pink1 and Parkin have been shown to play integral roles in mitochondrial clearance by selectively targeting dysfunctional or damaged mitochondria to the autophagic pathway^24,25^. Hence, we examined whether Pink1/Parkin-mediated mitophagy participates in the protective effect of MSCs on mitochondrial fitness under HG conditions. HG reduced Pink1 and Parkin protein levels in a dose-dependent and time-dependent manner in HUVECs (Supplementary Figure S3A-F). Consistently, Pink1 and Parkin mRNA levels were decreased after exposure to HG but reversed by MSCs treatment (Supplementary Figure S3G and H), indicating that the decrease of Pink1 and Parkin protein levels may be due to the transcriptional repression. Moreover, cell fractionation experiments revealed that very little Pink1 co-sedimented with mitochondria under HG conditions in parallel with a decline in LC3-II expression; however, these phenomena were reversed by MSCs administration (Figs. 4a–c), suggesting an improvement of mitophagy. To further investigate whether MSCs rescue HG-induced mitophagy deficiency through the Pink1/Parkin-mediated mitophagy pathway, we evaluated the role of Pink1 using a genetic approach. Cells transfected with Pink1 small interfering RNA (siRNA) had lower protein levels of LC3-II under normal glucose conditions. Importantly, MSCs treatment restored the autophagy capacity in cells transfected with control siRNA but failed to do so in cells transfected with Pink1 siRNA (Figs. 4d–f). In addition, colocalization of LC3 and COXIV was reduced upon shifting to HG but preserved by treatment with MSCs. Nevertheless, siRNA targeting Pink1 canceled the MSCs-mediated restoration of colocalization (Figs. 4g, h). Consistent results were also observed when we used a Parkin siRNA instead of a pink1 siRNA (Supplementary Figure S4A and B). These results demonstrate that MSCs improve mitophagy via the Pink1/Parkin-dependent pathway.

**Fig. 5:**
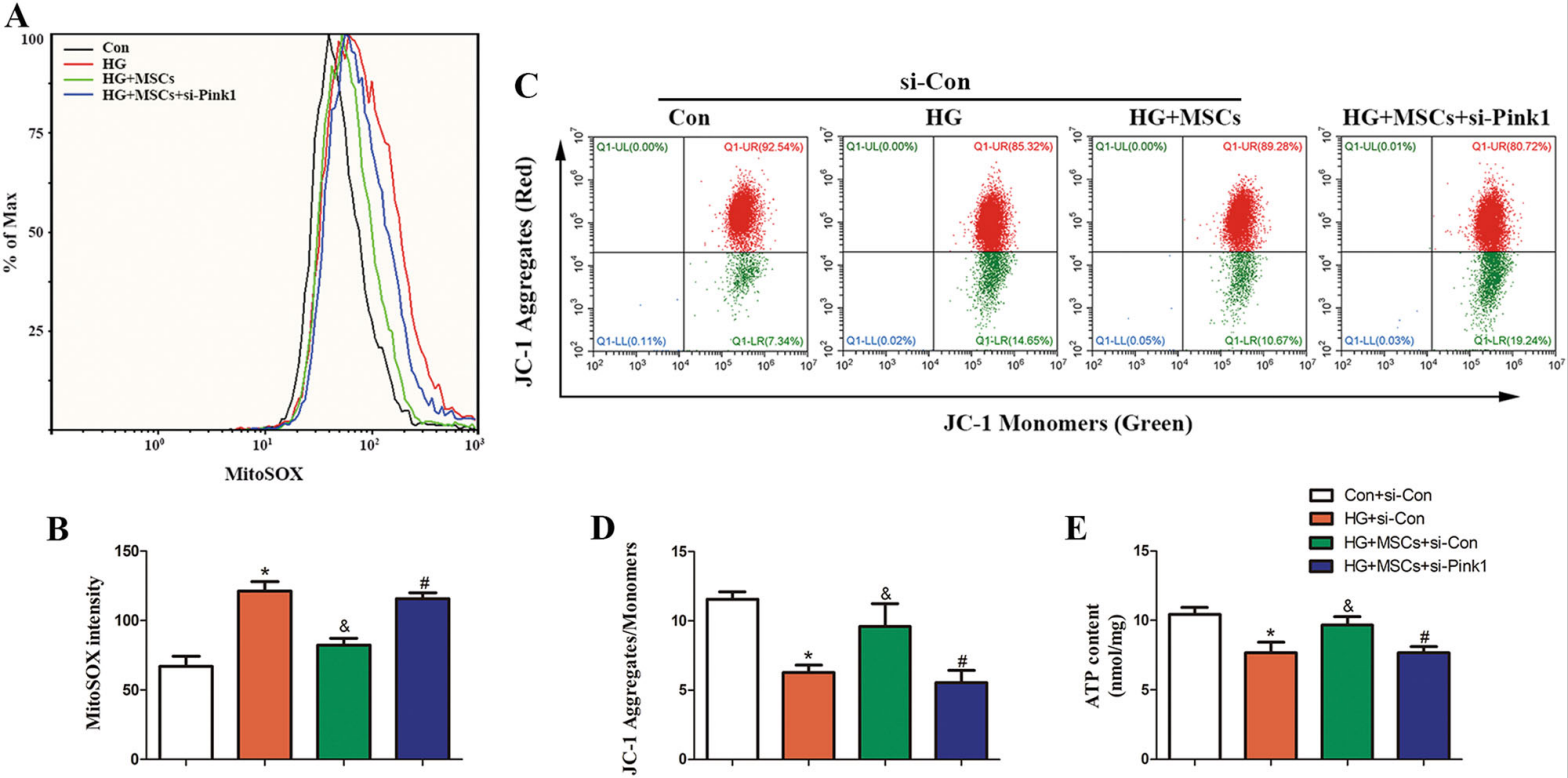
MSCs alleviate HG-induced mitochondrial dysfunction through Pink1-mediated mitophagy. **a**, **b** mtROS levels was determined by flow cytometry. **c**, **d** Mitochondrial membrane potential was detected by using the JC-1 staining. **e** ATP levels were quantified in HUVECs. Data are shown as the means ± SD from three independent experiments. (*P *<* 0.05 vs. Con/si-Con, ^&^*P* *<* 0.05 vs. HG/si-Con, ^#^*P* *<* 0.05 vs. HG/MSCs/si-Con)

We then determined the contribution of mitophagy to mitochondrial function in HUVECs. Overproduction of mtROS in response to HG was ameliorated by MSCs treatment, but this improvement was canceled when Pink1 was silenced (Figs. 5a, b). In addition, MSCs reversed the loss of Ψm (Figs. 5c, d, Supplementary Figure S5A and B) and enhanced ATP content (Fig. 5e) in response to HG depending on Pink1. Similar results were obtained when Parkin was knocked down (Supplementary Figure S4C), supporting the notion that Pink1/Parkin-mediated mitophagy underlies the protective effect of MSCs on mitochondria fitness.

**Fig. 6:**
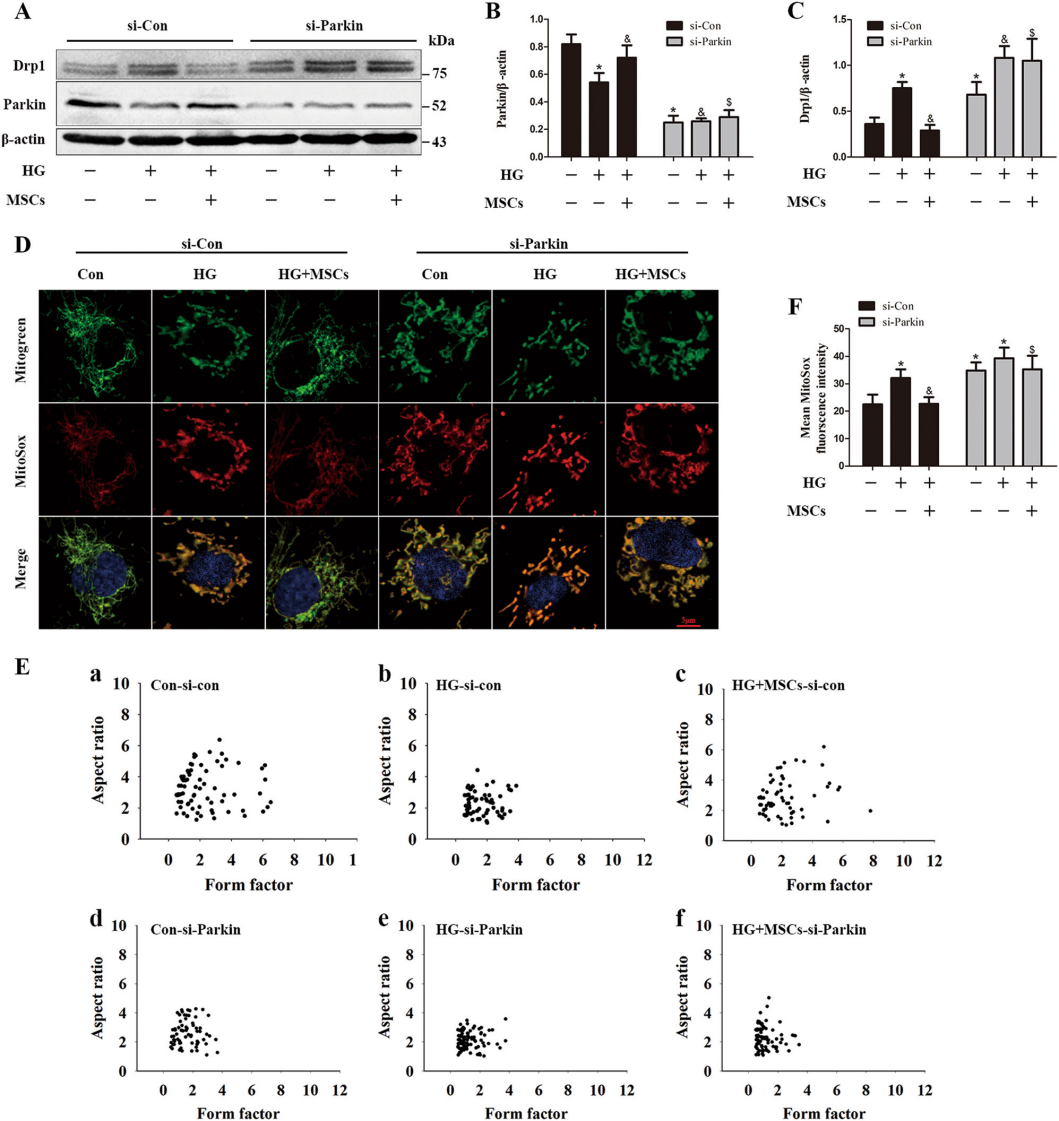
Abnormal mitochondrial morphology caused by HG can be rescued by MSCs through up-regulation of Parkin. **a**–**c** HUVECs transfected with control siRNA or siRNA targeting Parkin were analyzed by Western blot using antibodies against Drp1 and Parkin. **d** HUVECs were co-stained with MitoTracker green and MitoSOX Red to determine (**e**) mitochondrial morphology and (**f**) mtROS simultaneously. Data are shown as the means ± SD from three independent experiments. (*P *<* 0.05 vs. Con/si-Con, ^&^*P* *<* 0.05 vs. HG/si-Con, ^$^*P* *<* 0.05 vs. HG/MSCs/si-Con)

Previous studies have indicated that Parkin is a regulator of Drp1that may inhibit mitochondrial fission through ubiquitination and thereafter degradation of Drp1^26^. Knocking down endogenous Parkin leads to excessive dysfunctional fragmented mitochondria^27,28^. Thus, to explore whether Parkin-dependent manipulation of mitochondrial dynamics is behind the protective effect of MSCs on mitochondrial morphology, we knocked down Parkin using siRNA. Parkin knockdown increased the expression of Drp1. Remarkably, accompanied by a restoration of Parkin expression, MSCs treatment decreased HG-induced accumulation of Drp1, whereas this effect vanished when Parkin was knocked down (Figs. 6a–c). Furthermore, down-regulation of Parkin abolished the ability of MSCs to reduce HG-induced mitochondrial fragmentation and mtROS generation (Figs. 6d–f). Collectively, these results indicate that MSCs may reduce mitochondrial morphological fragmentation and subsequent ROS overproduction via the Parkin-dependent down-regulation of Drp1 expression.

### MSCs ameliorate HG-induced endothelial injury through Pink1/Parkin-mediated mitophagy

**Fig. 7:**
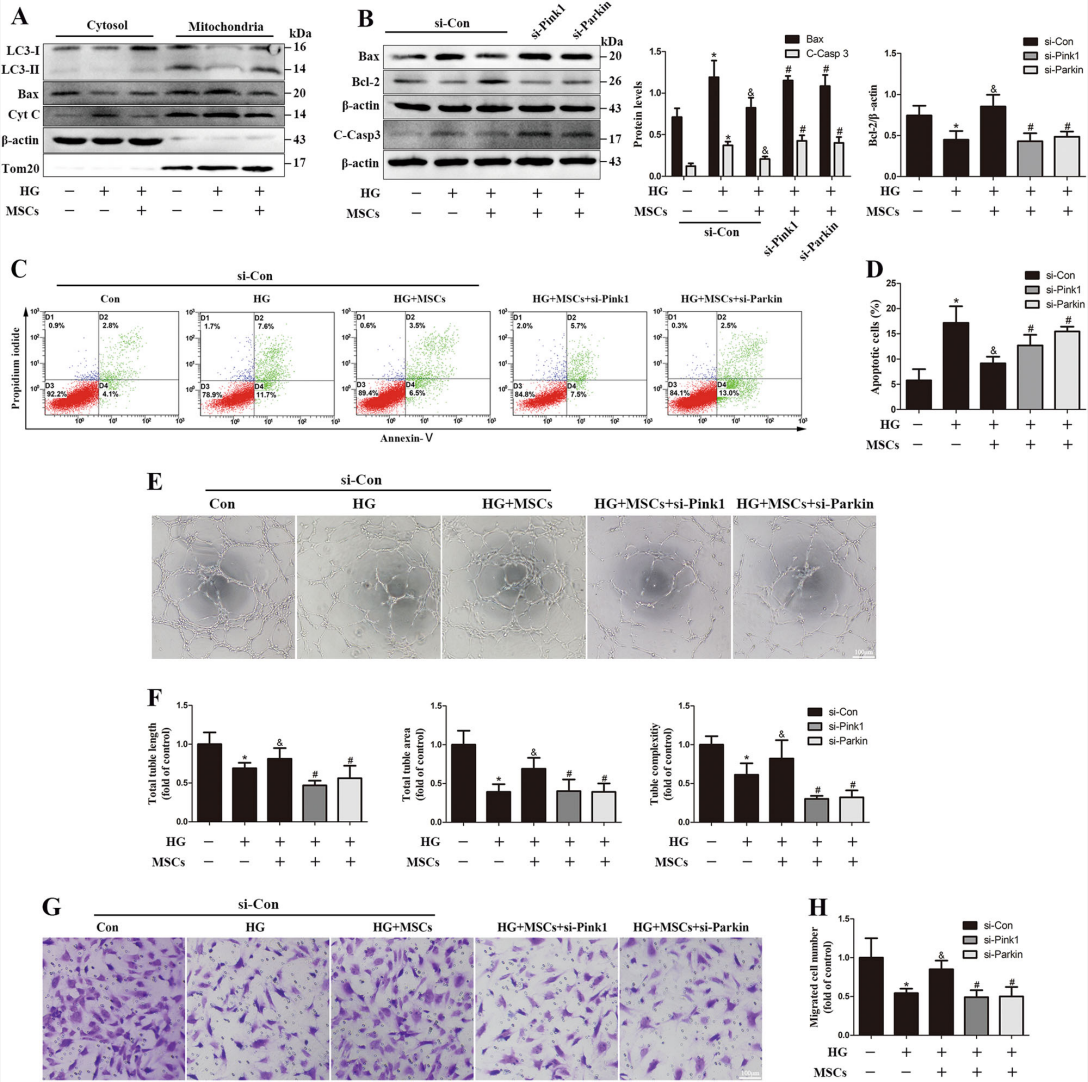
MSCs prevent HG-induced apoptosis and endothelial dysfunction via preserved Pink1/Parkin-mediated mitophagy. **a** Cytoplasmic and mitochondrial fractions from HUVECs were analyzed by Western blot, and the fractions were probed for LC3, Bax and Cyt C. **b** Western blot of whole-cell lysates probed for Bax, C-Casp3 and Bcl-2. **c**, **d** The apoptotic ratios of HUVECs in different groups were determined by flow cytometry. **e**, **f** Representative photomicrographs of tubules formed on matrigel. **g**, **h** Representative images and quantitative analysis of cell migration with a transwell assay in HUVECs. Data are shown as the means ± SD from three independent experiments. (*P *<* 0.05 vs. Con/si-Con, ^&^*P* *<* 0.05 vs. HG/si-Con, ^#^*P* *<* 0.05 vs. HG/MSCs/si-Con)

To explore whether improved mitophagy participates in the process of MSCs in amelioration of HG-induced endothelial injury, cell fractionation experiments were used to determine the effect of mitophagy on the proapoptotic factors Bax and Cyt C. Upon stimulation with HG, LC3-II expression was significantly decreased in mitochondrial fractions, suggesting an inhibition of mitophagy. Furthermore, activated Bax translocated from the cytoplasm to the mitochondria, which was accompanied by Cyt C release to the cytosol. However, MSCs treatment restored the expression of LC3-II and inhibited Bax and Cyt C redistribution (Fig. 7a), implying that the preserved mitophagy achieved by MSCs treatment may be involved in the prevention of the mitochondrial apoptotic pathway. To further confirm the protective effect of Pink1/Parkin-mediated mitophagy against HG-induced apoptosis, we inhibited mitophagy with Pink1- or Parkin-specific siRNA. MSCs treatment markedly reduced the expression of proapoptotic factor Bax and cleaved Caspase-3 (C-Casp3), but increased the anti-apoptotic molecule Bcl-2 compared with those of HG group, whereas either Pink1 or Parkin knockdown abrogated the anti-apoptotic effect of MSCs in HG conditions (Fig. 7b). Consistent results were also observed when analyzed with flow cytometry (Figs. 7c, d).

We next examined the effect of MSCs treatment on endothelial function in HG conditions. Angiogenesis analysis revealed that HUVECs formed less-developed tubule networks upon HG stimulation, with significant reductions in the length and area of tubules as well as the a decline in complexity. Coculture with MSCs improved angiogenic capacities; however, this beneficial effect was blunted with Pink1 or Parkin knockdown (Figs. 7e, f). Furthermore, siRNA targeting Pink1 or Parkin also suppressed the ability of MSCs to improve the migration under HG conditions (Figs. 7g, h). Taken together, these results demonstrate that Pink1/Parkin-mediated mitophagy is essential for MSCs to protect HUVECs against HG-induced apoptosis and endothelial dysfunction.

### Infusion of MSCs prevents the decrease of mitophagy in aortas from diabetic rats

Six months after diabetes was induced, diabetic rats exhibited severe hyperglycemia and body weight loss accompanied by degeneration of renal function as evidenced by blood urea nitrogen (BUN) (Supplementary Figure S6A-D). In addition, dyslipidemia with higher triglycerides, cholesterol, and LDL was observed in diabetic rats, but no significant difference was detected in HDL (Supplementary Figure S6E-H). Diabetic rats treated with MSCs exhibited a slight reduction in blood glucose levels and a notable increase in body weight. Furthermore, MSCs treatment also attenuated the BUN and triglycerides in diabetic rats but without significant effect on cholesterol, LDL, and HDL.

**Fig. 8:**
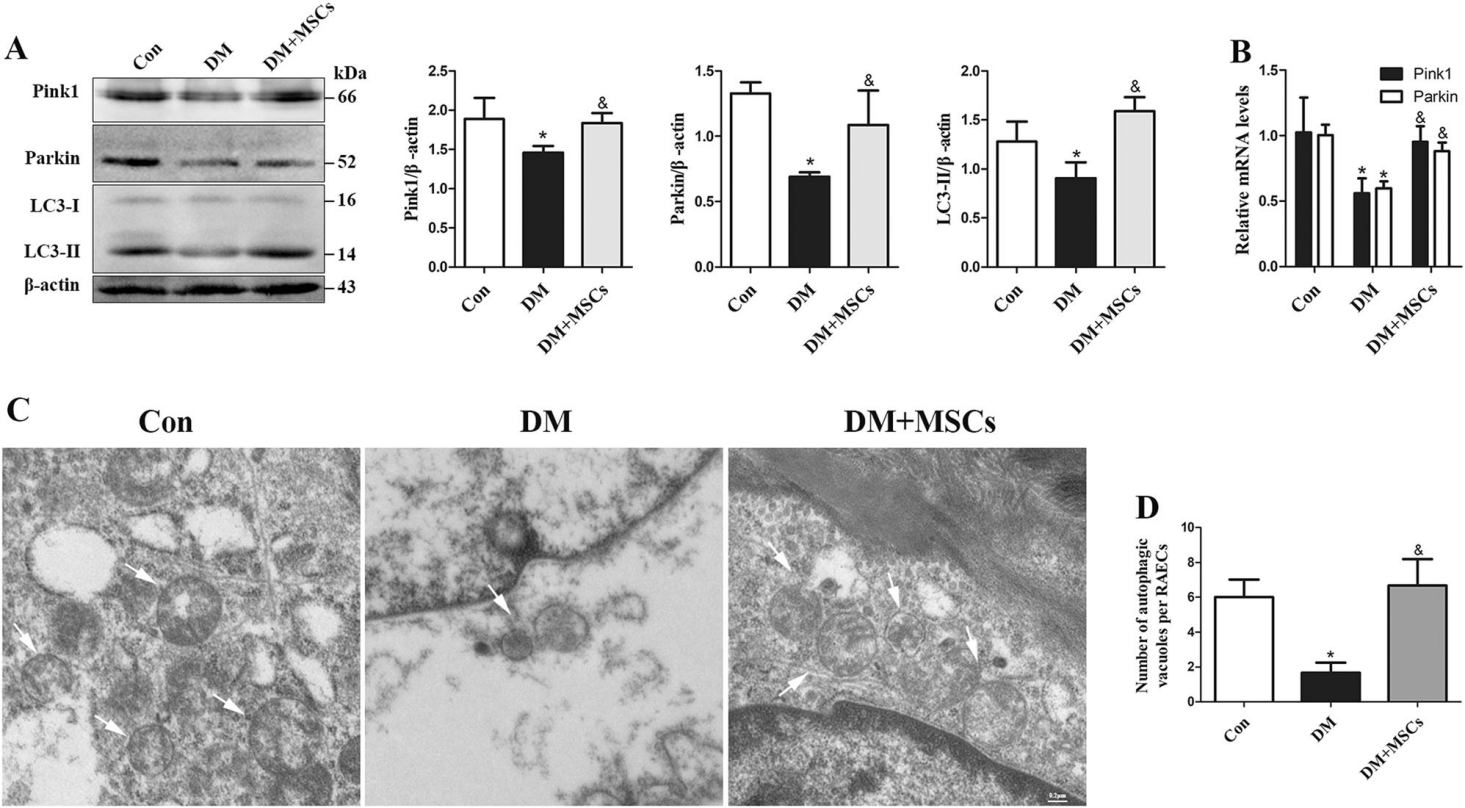
Infusion of MSCs preserves mitophagy in diabetic aortas. **a** Western blots of whole-aorta lysates probed for Pink1, Parkin, and LC3. **b** The mRNA expression levels of Pink1 and Parkin were determined by qPCR. **c**, **d** Ultrastructural images of autophagic vacuoles (white arrows) in rat aortic endothelium. Data are shown as the means ± SD from three independent experiments. (*P < 0.05 vs. Con, ^&^*P* < 0.05 vs. DM)

To assess the effect of MSCs therapy on mitophagy, we measured the expression of LC3-II, Pink1 and Parkin in rat aortas. MSCs treatment markedly increased the expression of these mitophagy-related proteins in diabetic rat aortas (Fig. 8a). Consistently, Pink1 and Parkin mRNA levels were decreased in diabetic rats but reversed by MSCs treatment (Fig. 8b). Furthermore, consistent results were observed when en face immunofluorescence staining was performed on rat aortas (Supplementary Figure S7A-F). Transmission electron microscopy (TEM) was applied to visually determine the status of autophagic vacuoles in RAECs. While there were fewer autophagic vacuoles in the diabetic group than in the controls, there were more in the MSCs-treated group (Figs. 8c, d).

### MSCs prevent mtROS overproduction, mitochondrial dysfunction and apoptosis in aortas from diabetic rats

**Fig. 9:**
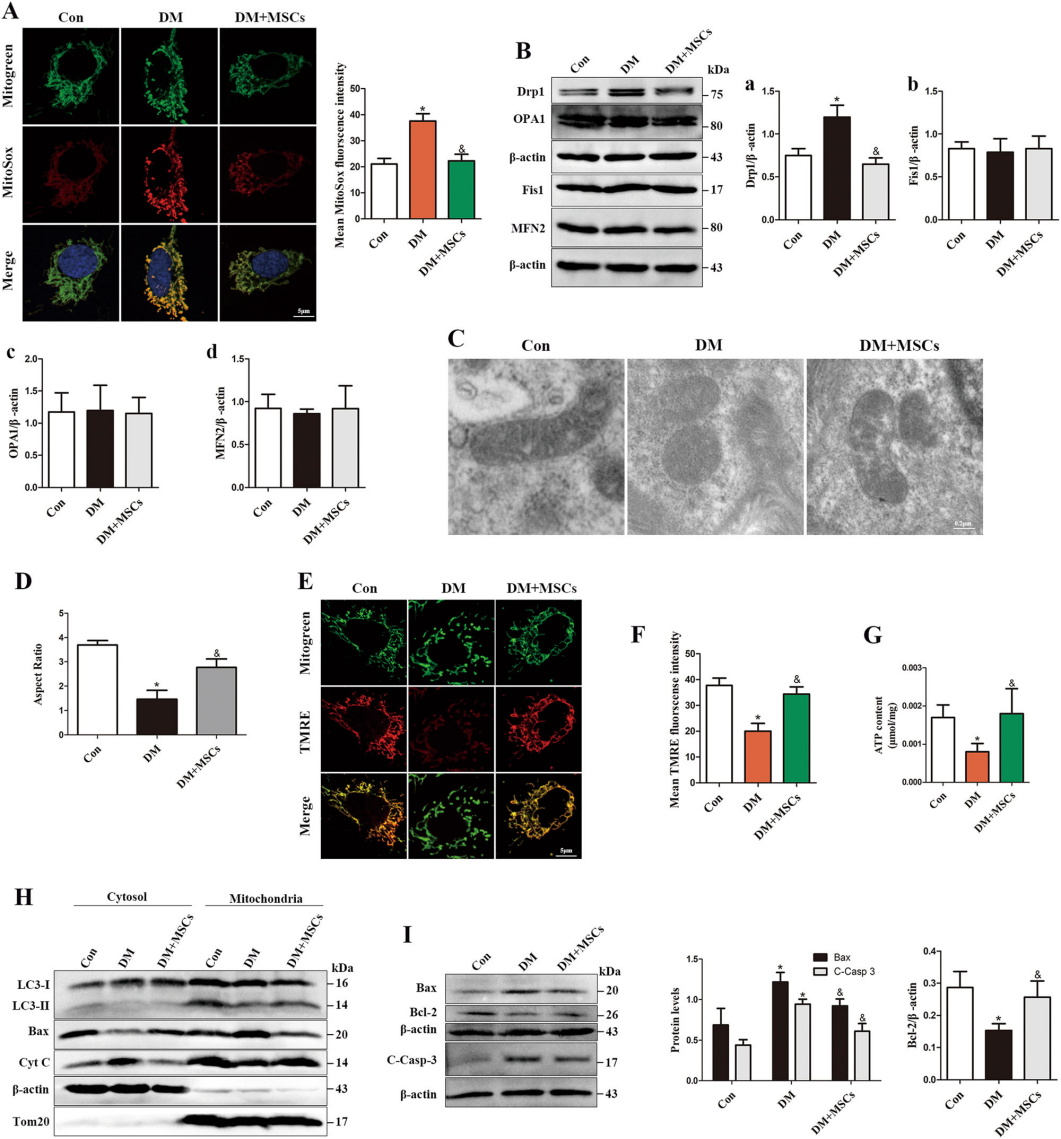
Infusion of MSCs attenuates mitochondrial dysfunction and apoptosis in diabetic aortas. **a** Representative fluorescent images showing mtROS generation by co-staining with MitoTracker green and MitoSOX Red in aortic endothelial cells isolated from control, diabetic rats and diabetic rats treated with MSCs. **b** Mitochondrial dynamics-related protein expression in the aorta was analyzed by Western blot. **c** Electron microscopy images of mitochondria in the rat aortic endothelium. **d** Quantification of mitochondrial aspect ratio in rat aortic endothelium. **e**, **f** Representative fluorescent images showing the mitochondrial membrane potential by co-staining with MitoTracker green and TMRE in aortic endothelial cells from the groups described in **a**. **g** ATP levels were quantified in the aorta for each group from **a**. **h** Cytoplasmic and mitochondrial fractions from rat aorta lysates were analyzed by Western blot, and the fractions were probed for LC3, Bax, and Cyt C. **i** Western blot of whole-rat aorta lysates probed for Bax, C-Casp3, and Bcl-2. Data are shown as the means ± SD from three independent experiments. (*P < 0.05 vs. Con, ^&^*P* < 0.05 vs. DM)

To specifically test the effect of MSCs treatment on hyperglycemia-induced mtROS overproduction in RAECs, mtROS levels were determined in primary RAECs isolated from rat aortas (Supplementary Figure S8A, B and C). Consistent with the results observed in cultured HUVECs, we observed a marked increase in mtROS generation from the isolated RAECs of diabetic rats, whereas this increase was prevented in diabetic rats treated with MSCs (Fig. 9a). In parallel, MSCs treatment inhibited hyperglycemia-induced increase in Drp1 levels in rat aortas (Fig. 9b, a) but exerted no effect on the expression of OPA1, MFN2 and Fis1 (Fig. 9b, b–d). Furthermore, TEM revealed that diabetic RAECs had fragmented sphere-shaped mitochondria compared with those of controls, whereas MSCs-treated diabetic rats revealed apparently elongated cylindrical-shaped mitochondria, as quantified by aspect ratio (Figs. 9c, d), indicating that mitochondrial morphology was restored. We also observed that, although hyperglycemia-induced a marked decrease in Ψm and ATP contents in diabetic rats (Figs. 9e–g), MSCs treatment notably relieved these alterations, suggesting an improvement in mitochondrial fitness.

Finally, we measured cell apoptosis in rat aortas. Cell fractionation experiments revealed that altered distribution of both Bax and Cyt C occurred in diabetic rats with concomitant down-regulation of mitophagy, as evidenced by the decline in LC3-II levels in mitochondria (Fig. 9h). Furthermore, the expression of pro-apoptotic protein Bax and C-Casp3 were increased in aortas from diabetic rats, whereas anti-apoptotic protein Bcl-2 was significantly decreased (Fig. 9i). However, these unfavorable manifestations were markedly reversed in MSCs-treated diabetic rats. Collectively, these data suggest that MSC treatment inhibited hyperglycemia-induced mtROS overproduction, improved mitochondrial fitness and reduced cell apoptosis in diabetic rat aortas.

## Discussion

Here, we revealed a novel mechanism by which MSCs ameliorate HG-induced endothelial injury. Our finding demonstrated that HG resulted in mitochondrial dysfunction and subsequent mtROS overproduction; concurrently, mitophagy was blocked, leading to an accumulation of damaged mitochondria and promoting mitochondrial compromise, which further accelerated the deterioration of mitochondrial dysfunction and triggered the mitochondrial apoptotic cascade, eventually causing endothelial injury. Administration of MSCs improved Pink1/Parkin-mediated mitophagy, which accelerated the clearance of impaired mitochondria and inhibited mtROS overproduction, consequently protecting endothelial cells from the mitochondrial apoptotic pathway.

Accumulating amounts of evidences indicate that mitochondrial fragmentation predominated by mitochondrial fission is a necessary component for HG-induced ROS overproduction^6,29^. Inhibition of mitochondrial fission has been shown to prevent mitochondrial dysfunction, ROS overproduction and subsequent endothelial dysfunction in mice or patients with diabetes mellitus^30–33^. In the current study, we observed excessive mitochondrial fragmentation and mtROS generation upon HG insult. By examining the expression of fission/fusion-related proteins, we found that only Drp1, but not other dynamics-related proteins, was up-regulated upon HG stimulation. Drp1 is essential for mitochondrial fission and has been shown to play a central role in mediating mitochondrial dysfunction and oxidative stress in diabetic nephropathy and diabetes-accelerated atherosclerosis^31,34^. MSCs treatment inhibited Drp1 expression and its translocation to the mitochondria. In addition, mitochondrial morphology was restored, and mtROS was reduced in parallel with enhancement of the Ψm and ATP production, suggesting a protective effect of MSCs on mitochondrial fitness.

Recently, mitophagy has emerged as a cytoprotective mechanism to maintain mitochondrial homeostasis and cell survival under conditions of stress^25^. Deficient mitophagy has been reported in podocytes of mice with diabetes^35^. Consistently, we found that mitophagy was markedly decreased in both our in vitro and in vivo studies, which was accompanied by mitochondrial dysfunction. MSCs treatment significantly improved mitochondrial fitness, whereas this beneficial effect was blunted when the mitophagy parthway was blocked by 3-MA, indicating the mitochondrial protection conferred by MSCs may partially dependent on mitophagy. The molecular events that orchestrate the selective recognition and removal of damaged mitochondria behind the process of mitophagy are now well elaborated. It is conceivable that the Pink1/Parkin-mediated mitophagy pathway may be implicated in the beneficial effect of MSC therapy. Evidence is accumulating for the relationship between mitophagy and diabetes. Improving Pink1/Parkin-mediated mitophagy protects tubular cells against severe oxidative stress in diabetic kidney disease^36^. Furthermore, Pink1 overexpression confers tubular cells the ability to counterbalance the damaging effects of HG-induced mitochondrial fragmentation, ROS overproduction and apoptosis^9^. Consistent results were observed in Parkin-overexpressing pancreatic β-cells^37^. Our current findings demonstrated that expression of Pink1 and Parkin were dramatically down-regulated under HG conditions and that mitophagy inhibition, mitochondrial compromise and cell apoptosis were present. MSCs treatment promoted the expression of these two mitophagy regulators, and improved mitochondrial fitness and endothelial dysfunction. However, these beneficial effects of MSCs were absent when either Pink1 or Parkin was knocked down, suggesting the protective effect of MSCs on endothelial cells may be at least partially regulated by Pink1/Parkin-mediated mitophagy. It has previously been demonstrated that Pink1 can interact with pro-autophagic protein Beclin1 and enhance autophagy^38^. Furthermore, autophagy was reported to be reduced in different cell model systems with Pink1 knockdown or knockout^39^. According to these reports, it seems possible that Pink1, in addition to mitophagy, has also a functional role in regulation of autophagy. Presumably, these two mechanisms may work together or in parallel during the process of MSCs in amelioration of HG-induced mitochondrial injury.

Several lines of evidence indicated that the Pink1/Parkin pathway plays an important role in modulating mitochondrial dynamics by suppressing fission-induced mitochondrial fragmentation^27,28,40^. Parkin was proved to ubiquitinate Drp1 and lead to its degradation^26,41^. Furthermore, a recently published report noted that UC-MSCs effectively limit cisplatin-induced tubular cell mitochondrial fragmentation^42^. In addition, one finding of the current study also revealed that MSCs treatment significantly inhibited the expression of Drp1 and its translocation to mitochondria as well as the concomitantly restoration of mitochondrial morphology. Therefore, these data motivated us to address the question of whether MSCs prevent HG-induced fragmentation and ROS overproduction through up-regulation the expression of Parkin. We found that Parkin deficiency resulted in an increase in Drp1 protein levels combined with impaired mitochondrial morphology and increased ROS generation. Importantly, this phenotype was more prominent under HG conditions. MSCs treatment attenuated HG-induced excessive mitochondrial fragmentation and ROS overproduction, whereas these protective effects were abolished with Parkin knockdown, supporting the notion that inhibition of Drp1 by Parkin may be an important mechanism by which MSCs protect against HG-induced fragmentation and subsequent ROS overproduction.

Given that DM is a chronic, progressive disease, we extended the exposure to hyperglycemic conditions to more closely mimic diabetic conditions. Correspondingly, multiple MSC infusions were performed to evaluate the effects of MSCs transplantation. Studies from type2 diabetic rats demonstrated that a single MSC injection ameliorated hyperglycemia for a short time, while multiple intravenous infusions of MSCs could reverse blood glucose level to a normal level^11,43^. However, inconsistent with these reports, we detected only a slight decrease in blood glucose levels after multiple infusions of MSCs, which is far from reaching a normal level. One possible interpretation for this observation may be that pancreatic β-cells are thoroughly destroyed in STZ-induced type1 diabetes. Furthermore, we previously showed that in type1 diabetic rats, short-term therapy with MSCs could effectively inhibit hyperglycemia-induced endothelial oxidative injury without any amelioration on glycemic control^18^. Thus, we speculate this long-term effect of MSCs on mitochondrial fitness may not depend on its mild glucose-lowering properties.

In summary, our results demonstrated that MSCs treatment attenuates hyperglycemia-induced endothelial injury through the mitophagy-mediated removal of dysfunctional mitochondria, in which the Pink1/Parkin pathway plays a critical role. Hence, this study provides novel insights into our understanding of MSC-based therapeutic mechanisms.

## Materials and methods

### Reagents and antibodies

Glucose (G8270), Mannitol (78513), CQ (C6628), and STZ (S0130) were purchased from Sigma. The siRNAs for Pink1 and Parkin, along with control siRNA and siRNA transfection reagent (human, sc-29528), were purchased from Ribobio Co., Ltd. Antibodies used for immunoblotting including rabbit anti-Parkin (ab15954); rabbit anti-Pink1 (ab23707) and rabbit anti-Fis1 (ab71498) were purchased from Abcam. Mouse anti-OPA1 (612606) was obtained from BD Biosciences. Mouse anti-Drp1 (14647); rabbit anti-LC3 (2775); rabbit anti-Tom20 (42406); rabbit anti-Bax (2772); rabbit anti-MFN2 (9482); rabbit anti-Cleaved Caspase-3 (9664); mouse anti-COXIV (11967) and rabbit anti-Cyt C (4272) were purchased from Cell Signaling Technology. Rabbit anti-β-actin (AC006) and rabbit anti-Bcl-2 (A2212) were obtained from Abclonal. Goat anti-CD31 (AF3628) was purchased from R&D Systems. Donkey Anti-Rabbit DyLight® 550 and Donkey anti-Goat Alexa Fluor 647 secondary antibodies for immunofluorescence were obtained from Abcam and Life Technologies, respectively.

### Cell isolation and culture

Human umbilical vein endothelial cells (HUVECs) were isolated from human umbilical cord veins as previously described^44^ and identified by flow cytometry (Beckman Coulter, Miami, FL, USA) with specific anti-CD31-PE and anti-CD144-FITC antibodies (BD, PharmingenTM, San Diego, USA). Endothelial cells were cultured in supplemented ECM-2 basal media (Millipore Corporation, Billerica, MA, USA) in a humidified atmosphere of 5% CO_2_/95% air at 37 °C. As control cell, human skin fibroblast cells (HSFs) were originally isolated by collagenase digestion of excised skin fragments as previously described and maintained in low-glucose DMEM (Gibco, Grand Island, NY, USA) containing 10% FBS (Gibco)^45^. Human Umbilical Cord-MSCs (hMSCs) were provided by Sichuan Neo-Life Stem Cell Biotech Inc. and cultured in low-glucose DMEM (Gibco, Grand Island, NY, USA) containing 10% FBS (Gibco). Cells between passages 3 and 6 were used in all experiments reported herein.

### Coculture and treatment

The effects of HG on endothelial cell function were investigated by incubating with control (5.5 mmol/L) or HG (30 mmol/L) medium as previously described^21^. Osmotic control groups (to account for medium hyperosmolarity) were exposed to mannitol (24.5 mmol/L) in ECM-2 medium containing glucose (5.5 mmol/L). For co-culture experiments, hMSCs or HSFs were seeded in a transwell insert at a density of 1.0 × 10^4^/cm^2^ and cultured in complete low-glucose DMEM medium for 12 h before coculture with HUVECs. Prior to coculture, hMSCs or HSFs were washed with PBS three times and cocultured with HUVECs (ratio 1:3) in ECM-2 media with a final glucose concentration of 30 mmol/L.

### Western blot analysis

HUVECs and rat aortas were homogenized in lysis buffer with phenylmethanesulfonyl fluoride, and protein concentrations were measured by the BCA Protein Assay Kit (Beyotime Biotechnology). Aliquots of cell extracts containing 30–60 μg of protein were prepared in SDS-sample buffer, subjected to SDS-PAGE and transferred to polyvinylidene difluoride membranes (Millipore Corporation, Billerica, MA, USA). After blocking with 5% nonfat milk for 1 h, membranes were probed with primary antibodies overnight at 4 °C, followed by incubation with horseradish peroxidase-conjugated secondary antibodies. Then, the proteins were visualized by enhanced chemiluminescence (Amersham, UK) reagents in the Molecular Imager Gel Doc XR System (Bio-Rad, Hertfordshire, UK). The blots were analyzed with densitometry using Image J software (NIH, Bethesda, MD, USA).

### Mitochondrial morphology assessment

For the live cell imaging, mitochondria morphology was detected by staining with 100 nmol/L MitoTracker Green or 150 nmol/L MitoTracker Deep Red (Life Technologies,Carlsbad, CA) and examined by confocal microscopy (Nikon A1, Nikon Corporation, Japan). FF was defined as (perimeter^2^/4 *π* area). AR was determined as the ratio between the major and minor axes of the ellipse equivalent to the mitochondrion. Both parameters have a minimal value of 1 if the particle is a small perfect circle and the values increase as it becomes longer. AR represents mitochondrial length and increases in FF indicate increases in mitochondrial complexity^29^. Images were quantitated and analyzed by Image J software as previously described^46^.

### Mitochondrial isolation

Mitochondria were isolated from HUVECs or whole rat aorta using the Cell Mitochondria Isolation Kit (Beyotime Institute of Biotechnology, China) and the Tissue Mitochondria Isolation Kit (Beyotime Institute of Biotechnology, China), respectively. Briefly, samples were placed in ice-cold mitochondria isolation buffer and homogenized using a Douce homogenizer (Kontes Glass Co.) The homogenate was centrifuged at 600×*g* for 10 min at 4 °C. The supernatant was collected and then centrifuged at 11,000×*g* for 10 min at 4 °C to pellet the mitochondria, which were resuspended for analyses. After mitochondria collection, the supernatants were collected and centrifuged again at 12,000×*g* for 10 min at 4 °C. The supernatants were collected as the cytosol fraction. The total protein concentration of the isolated mitochondria and cytosol fraction was determined by the BCA Protein Assay Kit (Beyotime Biotechnology).

### Mitochondrial membrane potential

The mitochondrial membrane potential (Ψm) was detected by using TMRM (Life Technologies, Carlsbad, CA) and JC-1 (AAT Bioquest, Sunnyvale, CA, USA), respectively, according to the manufacturer’s instructions. For the measurement of Ψm and visualization of mitochondria, cells were washed twice with PBS and incubated with 50 μM TMRM and 100 nM MitoTracker Green at 37 °C for 30 min, after which the cells were washed twice with PBS and then observed under confocal microscope. For JC-1 experiments, cells were washed twice in PBS and stained with JC-1 dye for 30 min at 37 °C. Then, the cells were washed with PBS again and analyzed by flow cytometry.

### mtROS determination

mtROS production was measured with a flow cytometer or confocal microscope. For the flow cytometry analysis, cells were washed twice with PBS and loaded with 5 µM MitoSox for 30 min, after which the cells were washed twice with PBS and analyzed with flow cytometry. For fluorescence microscopy analysis, mtROS production was measured by co-staining with MitoSox (Invitrogen) and MitoTracker Green. Briefly, cells were washed twice with PBS, incubated with 5 µM MitoSox and 100 nM MitoTracker Green for 30 min, and washed twice with PBS. Fluorescence intensity was determined by confocal microscopy.

### Cell apoptosis assay

Cell apoptosis was analyzed using an Annexin V-FITC/PI staining kit (Roche Applied Science). After treatment, the cells were collected and labeled for 15 min with Annexin V and propidium iodide (PI). The apoptotic cells were detected via flow cytometry.

### ATP production

ATP levels were measured using the ATP Assay Kit (Beyotime Biotechnology) according to the manufacturer’s instructions. ATP level was presented as nmol/mg of protein.

### Immunofluorescence

Cells were washed in PBS, fixed with 4% paraformaldehyde in PBS for 10 min, permeabilized with 0.1% Triton X-100 in PBS for 10 min, and blocked in 1% BSA in PBS for 30 min. Primary antibodies were used at 1:300 for 1 h at room temperature, followed by secondary antibodies at 1:500 for 1 h at room temperature. Final washing included incubation with DAPI (Sigma-Aldrich) for 5 min. Cells were mounted with Fluoromount-G™ (eBioscience), and observed under a fluorescent microscope.

### In vitro angiogenesis analysis using matrigel

The matrigel assay was used to assess the spontaneous formation of capillary-like structures in vitro as previously described^18^. After treatment, HUVECs were plated at a density of 1.5 × 10^4^ cells/well in 96-well plates previously coated with growth factor-reduced matrigel matrix (BD Bioscience). After 6 h of incubation, tube formation was observed with a computer-assisted microscope.

### Migration assay

Cell migration assays were performed using 8.0 μm pore size Transwells (Corning, New York, NY, USA). After the treatment, 4 × 10^4^ HUVECs in ECM-2 serum-free medium were seeded in the upper chamber. Complete ECM-2 medium was placed in the lower chamber. After 12 h incubation at 37 °C, cells from the upper surface of the membranes were removed with a cotton swab, and cells that migrated to the lower surface were washed with PBS, fixed with 4% paraformaldehyde for 15 min and then stained with 0.1% crystal violet (Sigma, C6158) for 10 min. Migrated cells were then counted in four random microscopic fields.

### Animals and induction of diabetes

Eight-week-old male Sprague-Dawley rats from Chengdu Dossy Experimental Animal Co. Ltd. (Chengdu, China) with initial body weights of 200 g were used for the experiments. Before the experiments, the rats were housed for 1 week to adapt to the experimental animal facility with an ambient temperature of 22–25 °C, and were allowed access to water and food. Rats were then fasted for 12 h with free access to water, and diabetes was induced by a single dose of intraperitoneally injected STZ (55 mg/kg). Control rats received an equal volume of citric acid buffer. One week after the injection, rats with a fasting blood glucose (FBG) level ≥ 16.7 mM were considered diabetic. The diabetic rats were randomly assigned to the diabetic group or the diabetes with MSC treatment group. All animal protocols were reviewed and approved by the Animal Care and Use Committee of West China Hospital, Sichuan University.

### Isolation of bone marrow MSCs

To generate bone marrow MSCs, bone marrow mononuclear cells were harvested by flushing the tibiae and femurs of 3-week-old male Sprague-Dawley rats (50–55 g body weight) with saline. Cells were cultured in low-glucose DMEM containing 10% FBS at 37 °C in 5% humidified CO_2_ and identified as previously described^18^. MSCs between passages three and four were used for transplantation.

### Transplantation of MSC

Four weeks after the induction of diabetes, 4 × 10^6^ MSCs were suspended in 1 mL saline and injected into rats via the tail vein 10 times at 2-week intervals. Control diabetic rats were infused with 1 mL saline.

### En face immunofluorescence staining

Immunofluorescence staining of rat aortic ECs were performed as described previously^47^. Briefly, rats were anesthetized with pentobarbital sodium (30 mg/kg body weight). Then, one of the femoral arteries was cut to drain blood, and the circulatory system was perfused with saline containing 40 U/mL heparin through the left ventricle until the saline flowing out from the cut become clear, after which the circulatory system was perfused with prechilled 4% paraformaldehyde in PBS (pH 7.4) for 10 min. Subsequently, the whole aorta was dissected, stripped of fat and connective tissues carefully, cut open longitudinally, permeabilized with 0.1% Triton X-100 in PBS for 10 min and blocked with 5% BSA in Tris-buffered saline (TBS) containing 2.5% Tween-20 for 1 h at room temperature. Next, aortas were incubated with goat anti-CD31 and rabbit anti-Pink1, rabbit anti-Parkin or rabbit anti-LC3 in blocking buffer overnight at 4 °C. After rinsing with washing solution (TBS containing 2.5% Tween-20) 3 times, fluorescence-conjugated secondary antibodies (1:500 dilution, Donkey Anti-Rabbit DyLight® 550 and Donkey anti-Goat Alexa Fluor 647) were applied for 1 h at room temperature. Finally, after an additional 3 rinses in the washing solution, aortas were mounted with Fluoromount-G™. Aortas were examined by confocal microscopy with a 40 × lens.

### Transmission electron microscopy (TEM)

Fresh rat aortas were fixed in 2.5% glutaraldehyde solution and then dehydrated and embedded in Epon resin. Ultra-thin sections of embedded tissues were stained with 5% uranyl acetate and lead citrate solution and analyzed by TEM.

### Isolation of rat aorta vascular endothelial cells

Rat aortic endothelial cells (RAECs) were isolated as described previously^48^. Briefly, rats were anesthetized with pentobarbital sodium (30 mg/kg body weight). Saline containing 40 U/mL heparin was injected into the left ventricle to perfuse the aorta. Then, the aorta was quickly removed from the rats, and the attached adipose tissue and connective tissue were removed from the aorta. The aorta was cut into 1 mm rings. Each aortic ring was opened and seeded onto a dish coated with gelatin (0.1%), with the endothelium facing down. The segments were cultured in endothelial cell growth medium. When the endothelial cells sprouted from the aortic segment, the segments were removed, after which the cells were identified by flow cytometry and immunofluorescence staining with a specific anti-CD31 antibody.

### Quantitative real time RT-PCR (qPCR)

Real-time PCR reactions were performed in 96-well plates with a Bio-Rad CFX96TM Real-time PCR Detection System. Reactions were carried out in a final volume of 25 μl containing 12.5 μl 2 × QuantiFast SYBR Green PCR Master Mix (Qiagen), 500 nM of each primer (see Supplementary Table S1), 1 μl cDNA and 9.5 μl DNase/RNase-free water. The cycling program was 95 °C for 5 min to activate the polymerase followed by 40 cycles of denaturation at 95 °C for 10 s, annealing at 60 °C for 20 s and extension at 72 °C for 20 s. Melting curve analysis was conducted by heating samples from 65 to 95 °C with continuous fluorescent acquisition. All reactions were performed in triplicate for each cDNA sample. In addition, no template controls were tested for each primer pair. Standard curves were established using a 10-fold dilution series of purified PCR fragments as templates.

### Statistical analyses

All data are presented as the means ± SD from at least three independent experiments. Statistical analysis was performed by one-way ANOVA using the PROC ANOVA procedure of the SAS software package, version 9.0. Duncan’s multiple-range test was performed for multiple comparisons when one-way ANOVA results were significant. Differences of *P* *<* *0.05* were considered significant.

## Electronic supplementary material

Legends for Supplementary Figures:

Characteristics of HUVECs

HG induces mitochondrial impairment and apoptosis in HUVECs

HG inhibits Pink1 and Parkin expression in HUVECs

MSCs ameliorate HG-induced inhibition of mitophagy and mitochondrial dysfunction in a Parkin-dependent way

MSCs alleviate HG-induced decrease of mitochondrial membrane potential through Pink1-mediated mitophagy

Effects of MSCs on blood glucose, body weight, and lipid profiles in diabetic rats

Infusion of MSCs preserves mitophagy in diabetic rat aorta endothelial cells

Isolation and validation of rat aorta endothelial cells

Primers and relative information of target and reference genes
