# Supplementary material for: Mesenchymal stem cells ameliorate hyperglycemia-induced endothelial injury through modulation of mitophagy
Source: Cell Death Dis. 2018 Aug 6;9(8):837. doi: 10.1038/s41419-018-0861-x (PMC6078996; doi:10.1038/s41419-018-0861-x)
Supplement: Supplementary file 1 — Legends for Supplementary Figures: [file 41419_2018_861_MOESM1_ESM.docx]

**Legends for Supplementary Figures:**

**Figure S1.** **Characteristics of HUVECs.** (A) Microscopic views of HUVECs isolated from human umbilical cord veins from fresh cords. (B) Flow cytometric analysis of HUVECs P3 cultures showed abundant expression of the classical HUVECs markers CD31and CD144.

**Figure S2. HG induces mitochondrial impairment and apoptosis in HUVECs.**

(A) HUVECs were treated with different concentrations of glucose for 72 h, and cell lysates were analyzed by Western blot using an antibody against P62. (B) HUVECs were exposed to HG (30 mmol/L) for indicated times, and P62 protein levels were detected by Western blot. (C) Mitochondrial ROS was measured using MitoSox Red by flow cytometry. (D) Images of mitochondrial morphology visualized by MitoTracker Red staining of HUVECs; aspect ratios and form factor were quantiﬁed for each group. (E and F) HUVECs were treated with HG for 72 h, and mitochondrial dynamics-related protein expression was determined by Western blot. (G) Mitochondrial membrane potential was measured using the JC-1 probe by flow cytometry. (H) The apoptotic ratios of HUVECs in different groups were determined by ﬂow cytometry using FITC-annexin V/PI double staining. Data are shown as the means ± SD from three independent experiments. (**P* < 0.05 vs. Con, ^&^*P* < 0.05 vs. HG48h).

**Figure S3. HG inhibits Pink1 and Parkin expression in HUVECs.**

(A-C) Western blot analysis and quantitative change of Pink1 and Parkin after exposure to different concentrations of HG for 72 h. (D-F) Western blot analysis of Pink1 and Parkin level at various time points after exposure to HG. (G and H) The mRNA expression levels of Pink1 and Parkin were determined by qPCR. Data are shown as the means ± SD from three independent experiments. (**P* < 0.05 vs. Con, ^&^*P* < 0.05 vs. HG).

**Figure S4. MSCs ameliorate HG**-**induced inhibition of mitophagy and mitochondrial dysfunction in a Parkin-dependent way.** (A and B) Colocalization between LC3 and COXⅣ was used as measure of mitophagy, PCC > 0.5 signify detection correlation. (C) ATP levels were quantiﬁed in HUVECs. Data are shown as the means ± SD from three independent experiments. (**P <* 0.05 vs. Con/si-Con, ^&^*P* < 0.05 vs. HG/si-Con, ^#^*P* < 0.05 vs. HG/MSCs/si-Con).

**Figure S5. MSCs alleviate HG-induced decrease of mitochondrial membrane potential through Pink1-mediated mitophagy.** (A and B) Mitochondrial membrane potential was detected by using the TMRE staining. Data are shown as the means ± SD from three independent experiments. (**P <* 0.05 vs. Con/si-Con, ^&^*P* < 0.05 vs. HG/si-Con, ^#^*P* < 0.05 vs. HG/MSCs/si-Con).

**Figure S6. Effects of MSCs on blood glucose, body weight, and lipid profiles in diabetic rats.** (A) Protocol for infusion of MSCs in the STZ-induced type 1 diabetic model (related to Figure 8). (B) Blood glucose level, (C)body weight, (D) [blood](file:///F:\%E5%BA%94%E7%94%A8%E7%A8%8B%E5%BA%8F\%E6%9C%89%E9%81%93%E8%AF%8D%E5%85%B8\Dict\7.5.2.0\resultui\dict\?keyword=blood)[urea](file:///F:\%E5%BA%94%E7%94%A8%E7%A8%8B%E5%BA%8F\%E6%9C%89%E9%81%93%E8%AF%8D%E5%85%B8\Dict\7.5.2.0\resultui\dict\?keyword=urea)[nitrogen](file:///F:\%E5%BA%94%E7%94%A8%E7%A8%8B%E5%BA%8F\%E6%9C%89%E9%81%93%E8%AF%8D%E5%85%B8\Dict\7.5.2.0\resultui\dict\?keyword=nitrogen) (BUN), (E) triglycerides (F), cholesterol , (G) LDL and (H) HDL in control rats, diabetic rats and diabetic rats treated with MSCs. Data are shown as the means ± SD from three independent experiments. (**P <* 0.05 vs. Con, ^&^*P* < 0.05 vs. DM).

**Figure S7. Infusion of MSCs preserves mitophagy in diabetic rat aorta endothelial cells.** (A-F) Representative immunoﬂuorescence images of LC3, Pink1 and Parkin in rat aortic endothelium. Endothelial cell morphology was shown by CD31 staining. Data are shown as the means ± SD from three independent experiments. (**P <* 0.05 vs. Con, ^&^*P* < 0.05 vs. DM).

**Figure S8.** **Isolation and validation of rat aorta endothelial cells.** (A) Rat aortic segment was isolated and seeded onto a gelatin (0.1%) coated dish with the endothelium facing down. The segment was cultured in endothelial cell growth medium. (B) Immunofluorescence staining and flow cytometric analysis of CD31 in rat aorta endothelial cells isolated from rat aortas.
