# Supplementary figures and images for: Mesenchymal stem cells ameliorate hyperglycemia-induced endothelial injury through modulation of mitophagy
Source: Cell Death Dis. 2018 Aug 6;9(8):837. doi: 10.1038/s41419-018-0861-x (PMC6078996; doi:10.1038/s41419-018-0861-x)

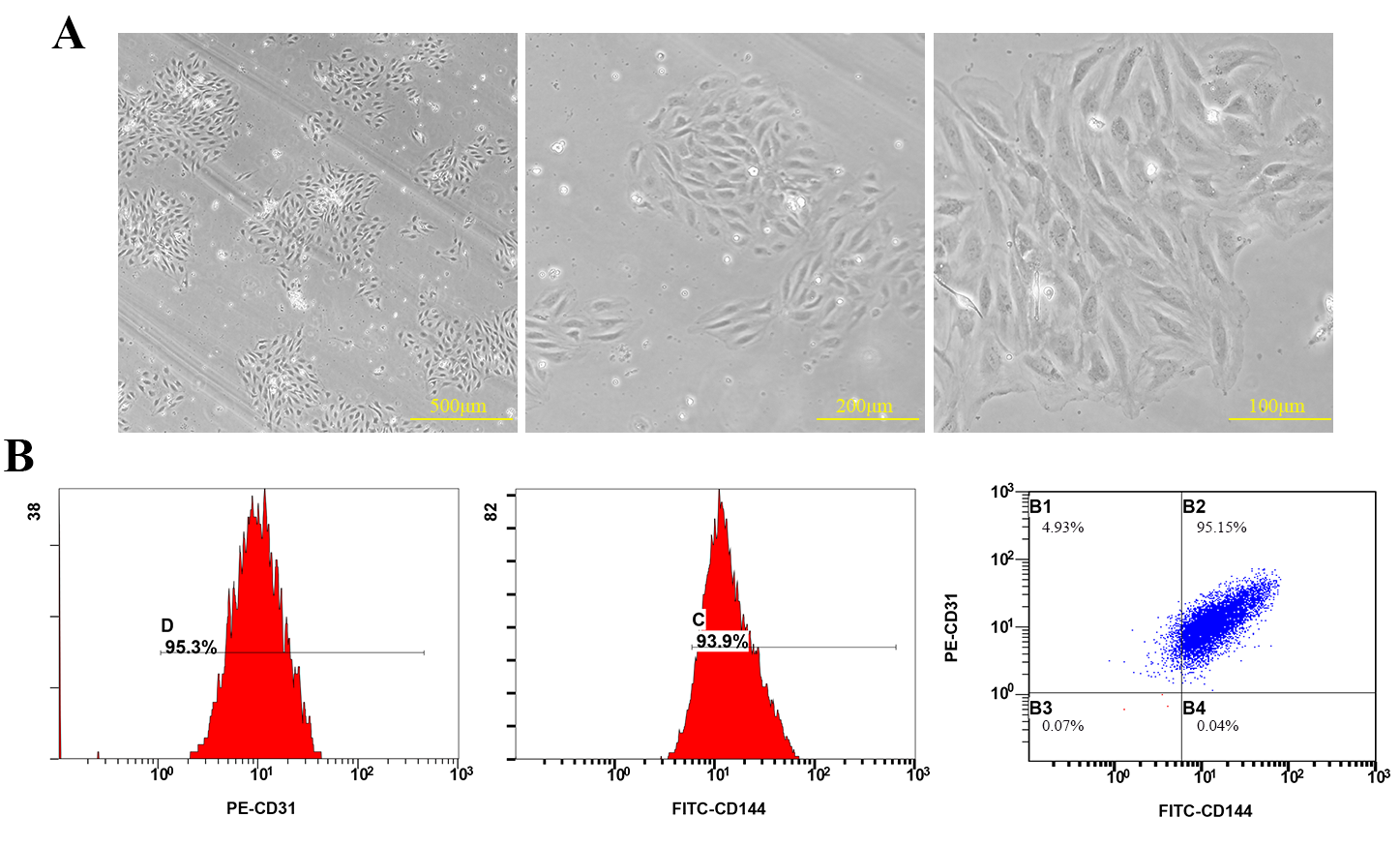

Supplement: Supplementary file 2 — Characteristics of HUVECs [file 41419_2018_861_MOESM2_ESM.tif]

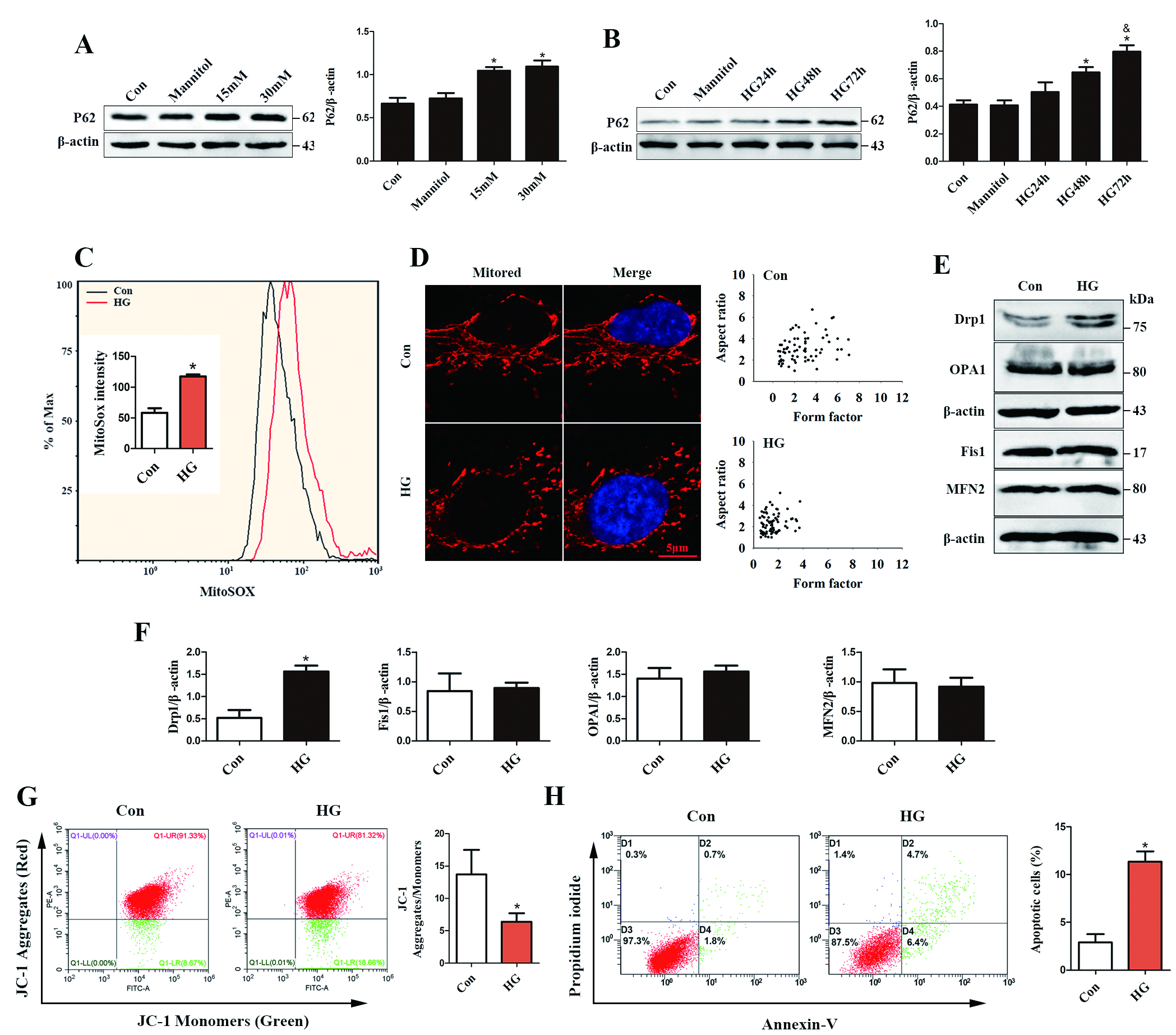

Supplement: Supplementary file 3 — HG induces mitochondrial impairment and apoptosis in HUVECs [file 41419_2018_861_MOESM3_ESM.tif]

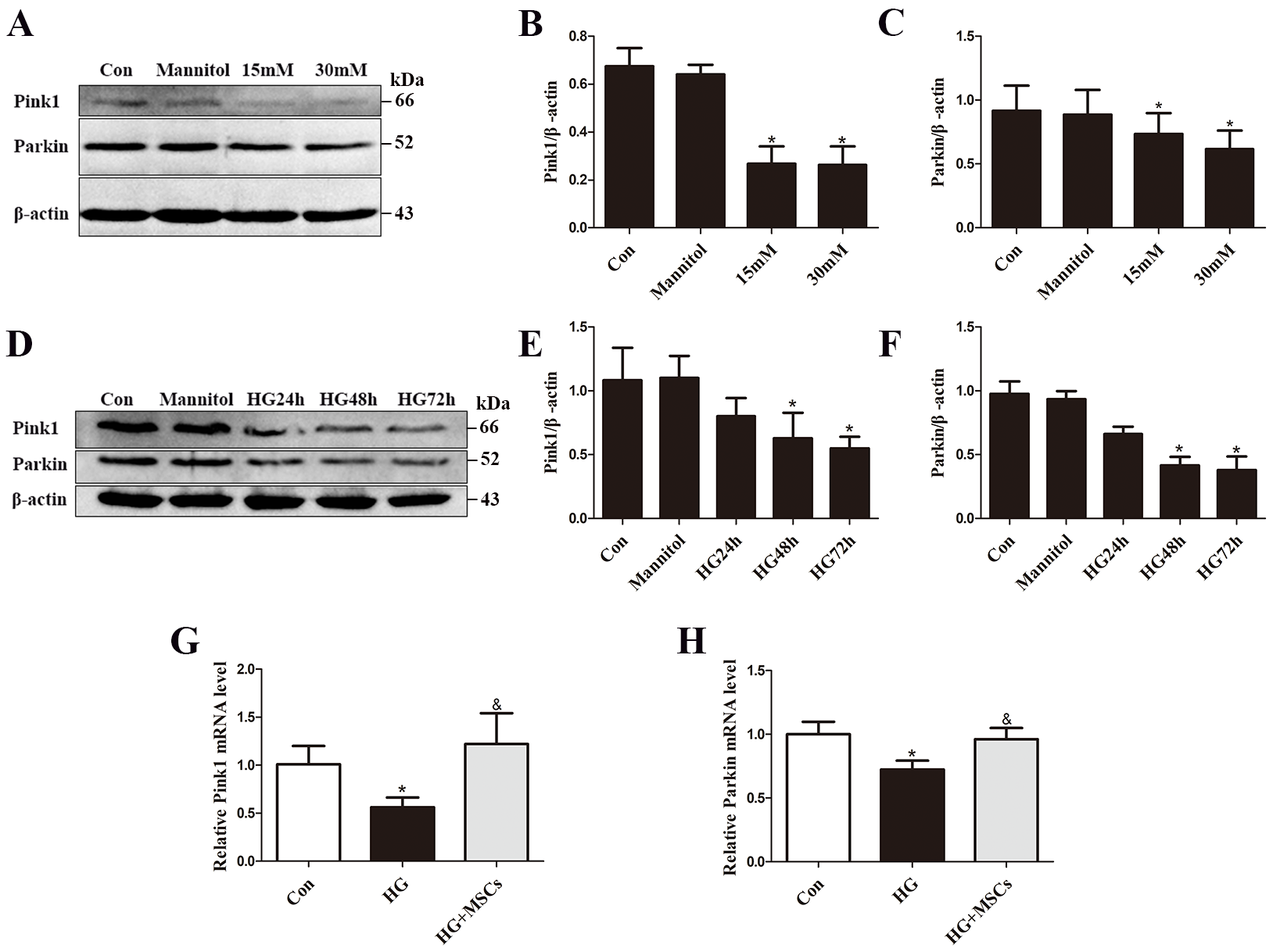

Supplement: Supplementary file 4 — HG inhibits Pink1 and Parkin expression in HUVECs [file 41419_2018_861_MOESM4_ESM.tif]

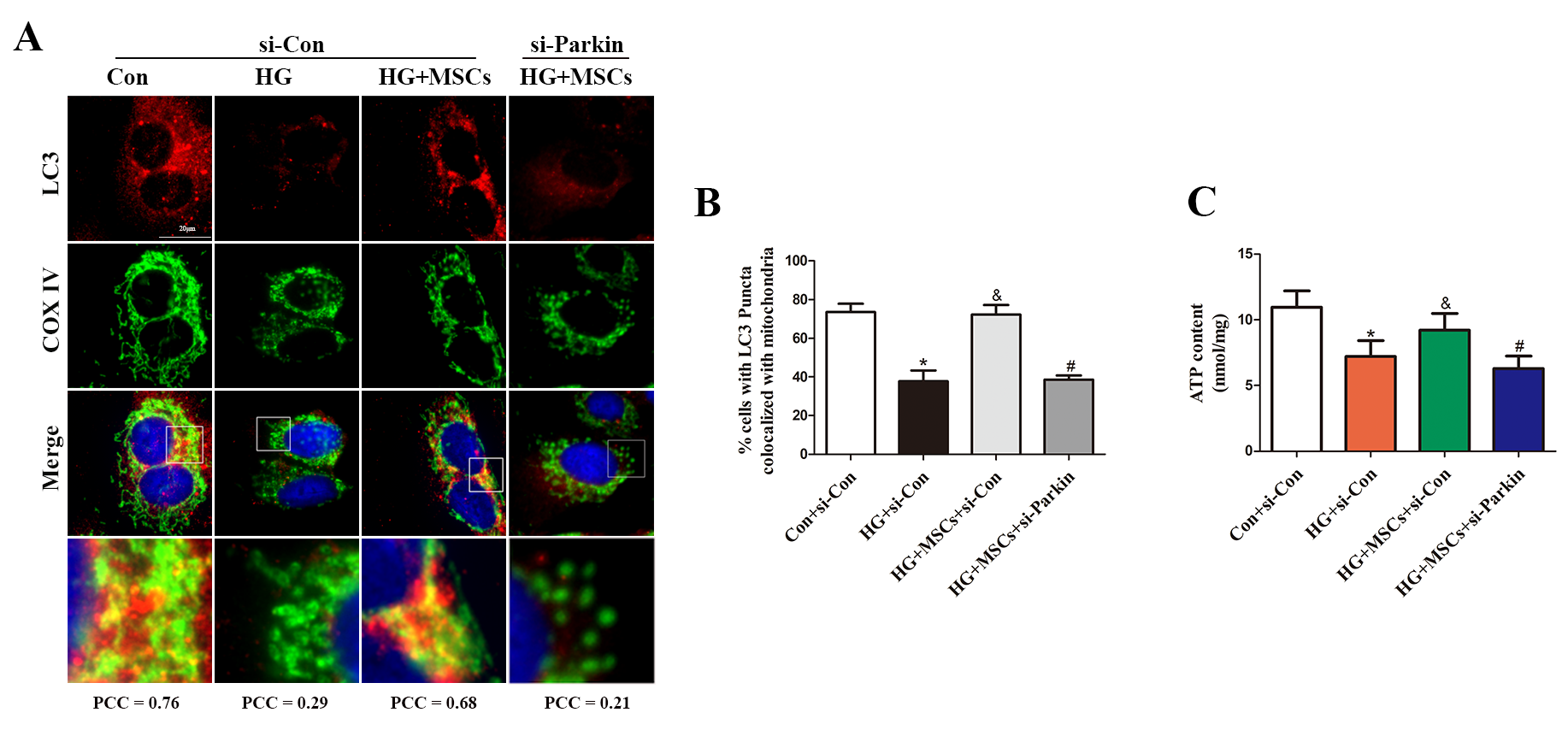

Supplement: Supplementary file 5 — MSCs ameliorate HG-induced inhibition of mitophagy and mitochondrial dysfunction in a Parkin-dependent way [file 41419_2018_861_MOESM5_ESM.tif]

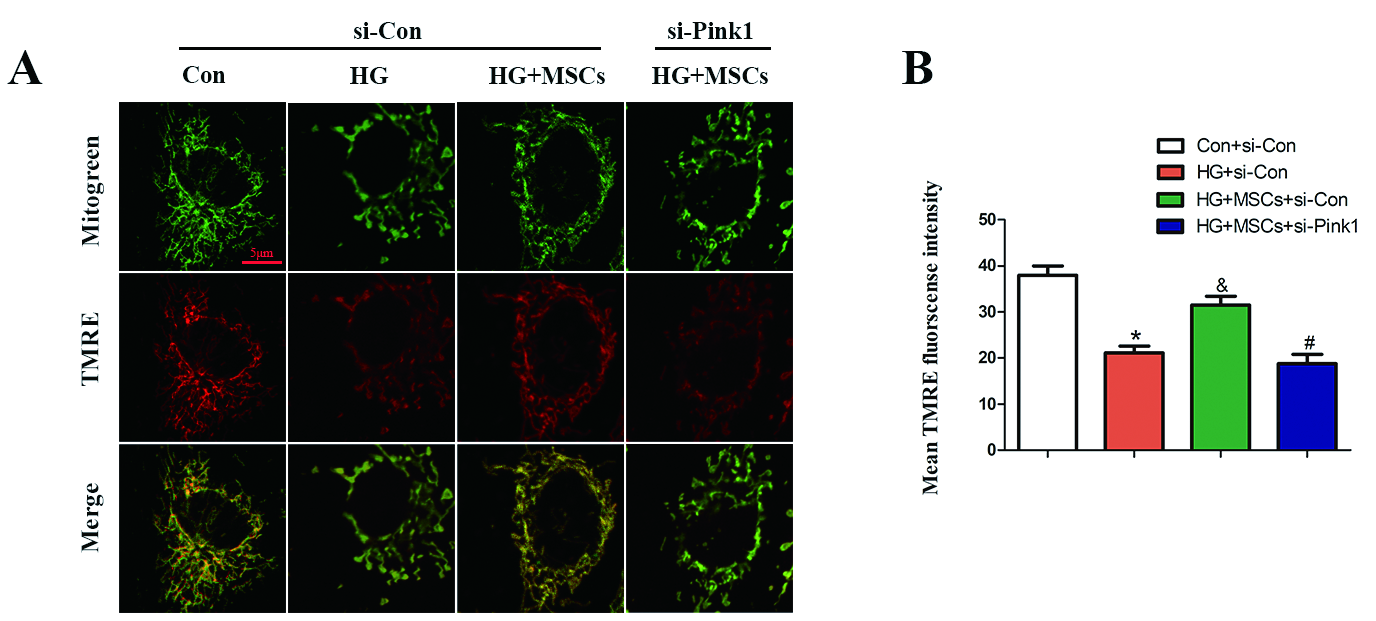

Supplement: Supplementary file 6 — MSCs alleviate HG-induced decrease of mitochondrial membrane potential through Pink1-mediated mitophagy [file 41419_2018_861_MOESM6_ESM.tif]

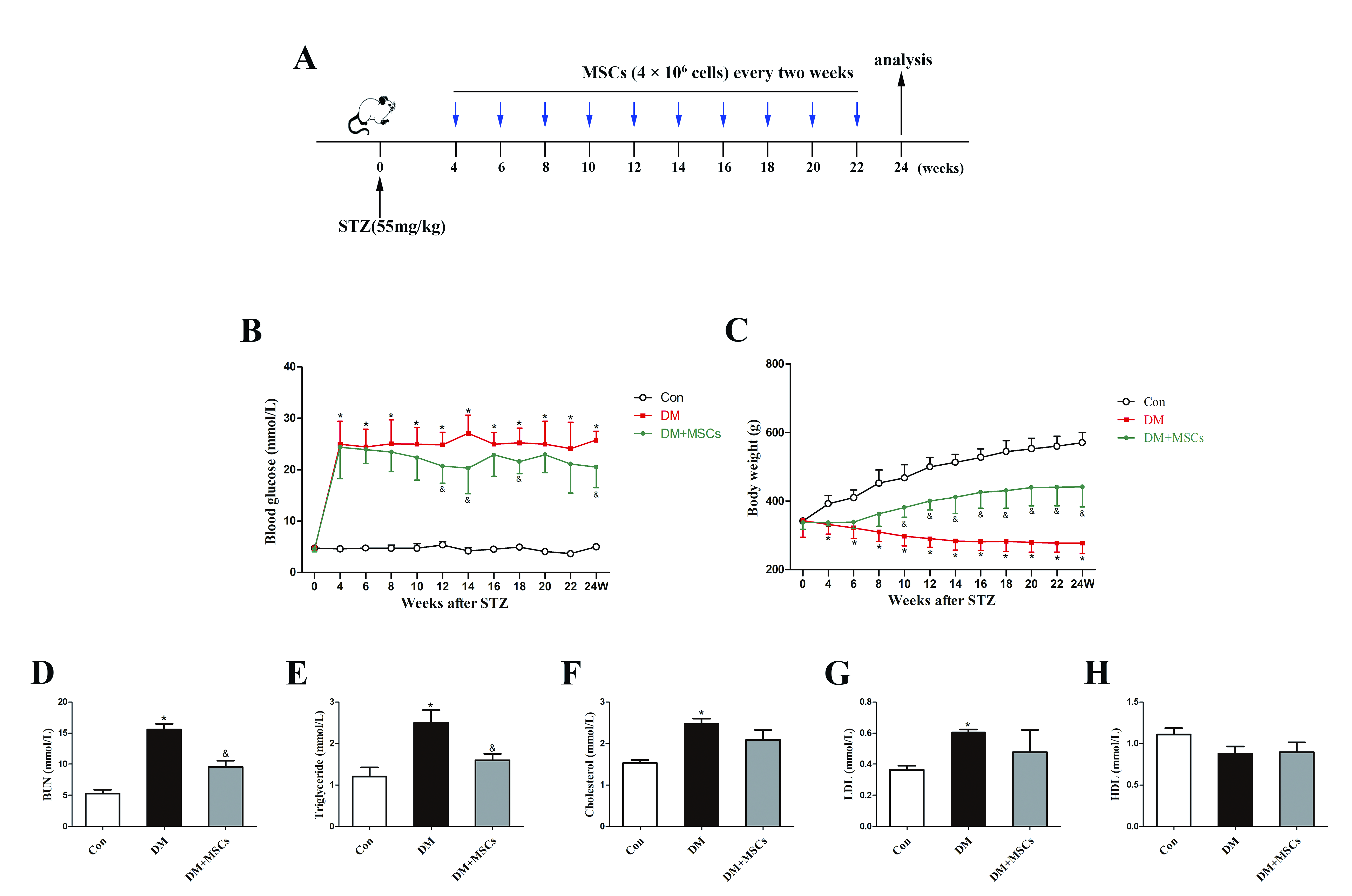

Supplement: Supplementary file 7 — Effects of MSCs on blood glucose, body weight, and lipid profiles in diabetic rats [file 41419_2018_861_MOESM7_ESM.tif]

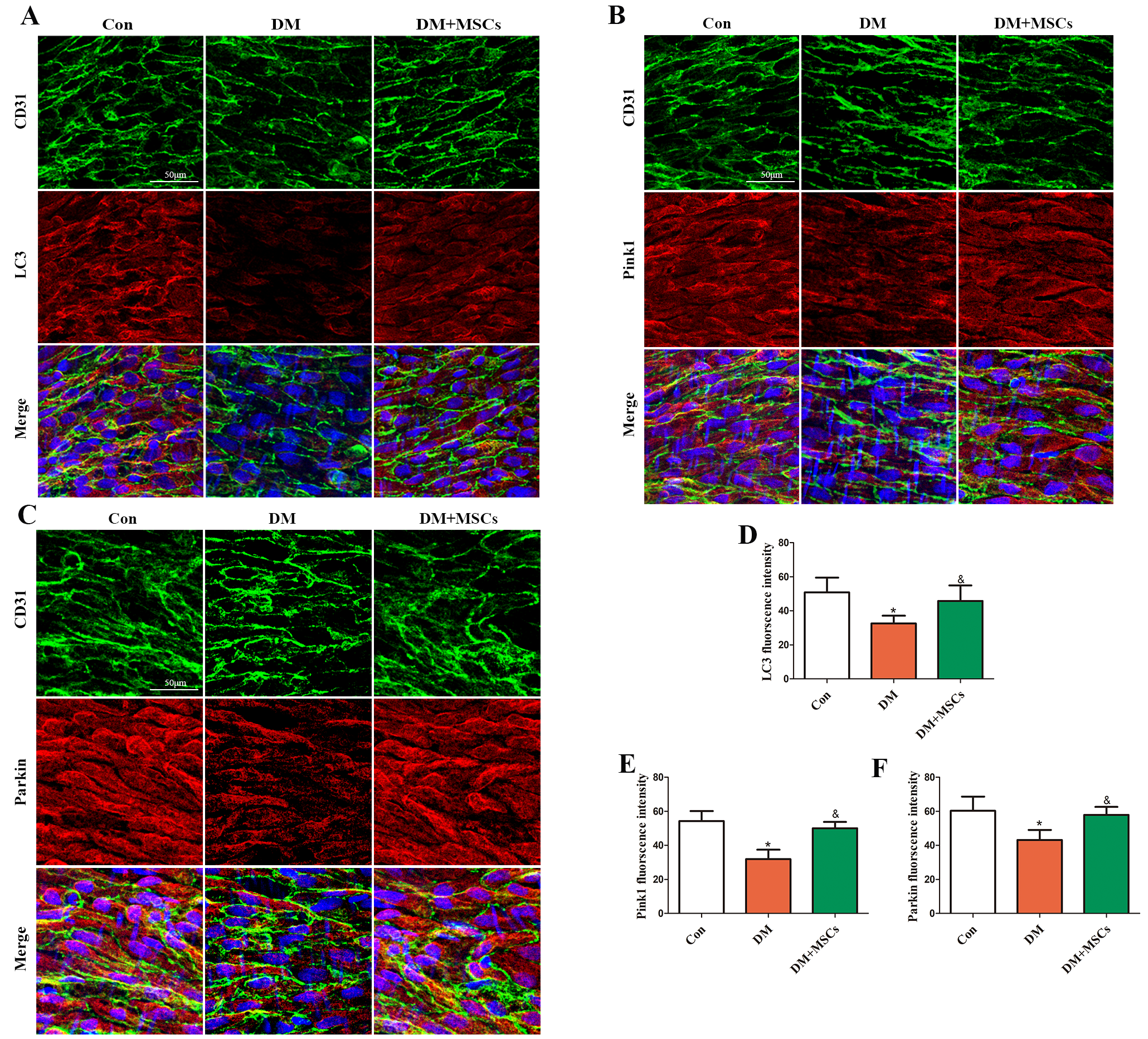

Supplement: Supplementary file 8 — Infusion of MSCs preserves mitophagy in diabetic rat aorta endothelial cells [file 41419_2018_861_MOESM8_ESM.tif]

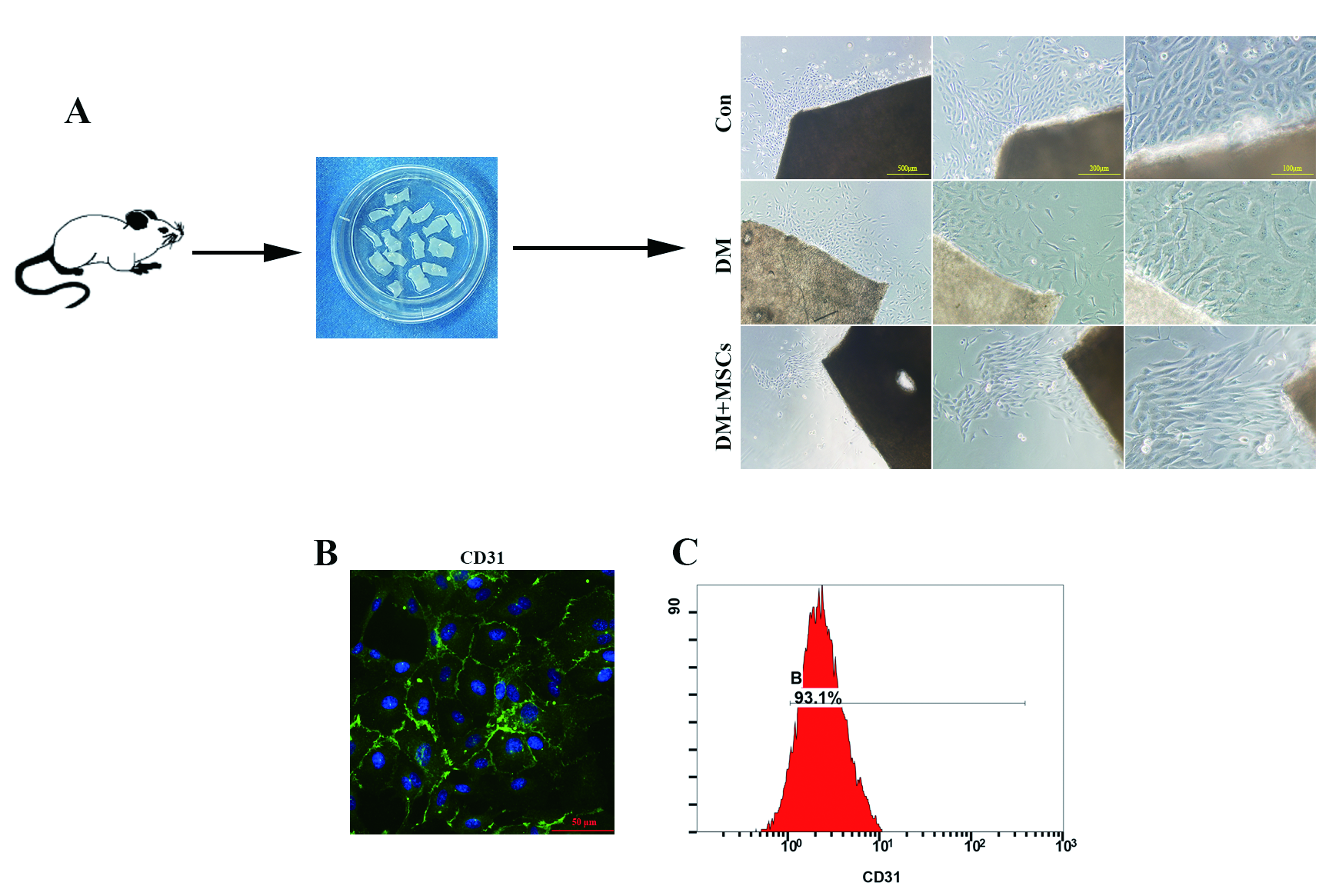

Supplement: Supplementary file 9 — Isolation and validation of rat aorta endothelial cells [file 41419_2018_861_MOESM9_ESM.tif]
