# Supplementary material for: Mesenchymal stem cells ameliorate hyperglycemia-induced endothelial injury through modulation of mitophagy
Source: Cell Death Dis. 2018 Aug 6;9(8):837. doi: 10.1038/s41419-018-0861-x (PMC6078996; doi:10.1038/s41419-018-0861-x)
Supplement: Supplementary file 10 — Primers and relative information of target and reference genes [file 41419_2018_861_MOESM10_ESM.docx]

| **Gene Symbol** | **Species** | **Primer sequence 5ˊto 3ˊ** | **Efficiency** | **R^2^** | **Slope** |
| --- | --- | --- | --- | --- | --- |
| *Pink1* | Human&Rat | F: CTGTCAGGAGATCCAGGCAATT  R: GCATGGTGGCTTCATACACAGC | 99.0% | 0.999 | -3.347 |
| *Parkin* | Human | F: GGAAGTCCAGCAGGTAGATCA R: ACCCTGGGTCAAGGTGAG | 97.8% | 0.999 | -3.377 |
| *β-actin* | Human | F: AGAGCTACGAGCTGCCTGAC R: AGCACTGTGTTGGCGTACAG | 98.7% | 0.999 | -3.354 |
| *Parkin* | Rat | F: CTGGCAGTCATTCTGGACAC R: CTCTCCACTCATCCGGTTTG | 95.3% | 0.998 | -3.44 |
| *β-actin* | Rat | F: GCAAATGCTTCTAGGCGGAC R: AAGAAAGGGTGTAAAACGCAGC | 99.6% | 0.999 | -3.333 |

Table S1. Primers and relative information of target and reference genes.
